# Lipidomic Profiling of the Olive (*Olea europaea* L.) Fruit towards Its Valorisation as a Functional Food: In-Depth Identification of Triacylglycerols and Polar Lipids in Portuguese Olives

**DOI:** 10.3390/molecules24142555

**Published:** 2019-07-13

**Authors:** Eliana Alves, Tânia Melo, Madalena P. Barros, M. Rosário M. Domingues, Pedro Domingues

**Affiliations:** 1 Mass Spectrometry Centre, Department of Chemistry & QOPNA & LAQV-REQUIMTE, University of Aveiro, Campus Universitário de Santiago, 3810-193 Aveiro, Portugal; 2 Department of Chemistry & CESAM & ECOMARE, University of Aveiro, Campus Universitário de Santiago, 3810-193 Aveiro, Portugal; 3 Cooperativa de Olivicultores de Nelas, C.R.L., Zona Industrial de Nelas, 3520-095 Nelas, Portugal

**Keywords:** mass spectrometry, lipidomics, glycolipid, glycosphingolipid, phospholipid, sphingomyelin, betaine, polyunsaturated fatty acid, hydroxy fatty acid, HPLC-MS/MS

## Abstract

Olives (*Olea europaea* L.) are classic ingredients in the Mediterranean diet with well-known health benefits, but their lipid composition has not been fully addressed. In this work, we characterised triacylglycerol (TAG) and polar lipid profiles of the olive pulp while using a complementary methodological approach that was based on solid-phase extraction to recover the neutral lipid (NL) and the polar lipid-rich fractions. The TAG profile was analysed in the NL-fraction by C_30_ reversed-phase liquid chromatography (LC) and the polar lipid profile by normal-phase hydrophilic interaction liquid chromatography (HILIC), with both being coupled to electrospray ionization-mass spectrometry (ESI-MS) and ESI-MS/MS. This approach identified 71 TAG ions that were attributed to more than 350 molecular species, with fatty acyl chain lengths from C11:0 to C26:0, including different polyunsaturated acyl chains. The polar lipids included 107 molecular species that belonged to 11 lipid classes that comprised phospholipids, glyceroglycolipids, glycosphingolipids, and betaine lipids. In addition to polyunsaturated fatty acids, some of the phospholipids, glycolipids, and glycosphingolipids that were identified in the olive pulp have been described as biologically active molecules. Lipidomic phenotyping of the olive pulp has led to the discovery of compounds that will allow for a better assessment of its nutritional value and new applications of bioactive lipid components in this functional food.

## 1. Introduction

Olives are the fruits of the olive tree (*Olea europaea* L.) and they are used for the production of table olives and olive oil, two classic ingredients of the Mediterranean diet. According to the International Olive Council, the world consumption of table olives in 2018 was ca. 2.7 million tons [1]. Portugal is the fourth European largest producer of tables olives, but their consumption in this country has decreased tenfold over the last three decades [1]. Amongst other factors, this is due to the common misconception that olives are high-calorie, high-fat foods, and that consumption should be moderate or avoided. On the contrary, olives are rich in oleic acid (C18:1*n*-9) and they contain various bioactive compounds that make them recognised as functional foods, contributing to the health benefits of the Mediterranean diet [2]. To date, the most important bioactive molecules that were found in table olives were phenolic compounds. These minor components were found to be up to 1700 mg caffeic acid per Kg of the edible part in table olives [3].

The olive pulp mainly contains water (70–75% of the pulp weight) and lipids (up to 30% in ripe olives, i.e., up to 300 g·Kg^−1^) [3,4]. The ripe olive fruit has ca. 92% of triacylglycerols (TAGs) [5] in the lipid pool. As such, lipids are the most abundant chemical components in this foodstuff, also being important for their nutritional value. The TAGs from the olive pulp contain oleic acid (about 73%), palmitic (C16:0), linoleic (C18:2*n*-6), stearic (C18:0), and α-linolenic (C18:3*n*-3) acids [6,7]. Monounsaturated fatty acids (MUFAs) and essential polyunsaturated fatty acids (PUFAs) are the most important nutritional elements of this oily fruit. The consumption of MUFAs, such as oleic acid, is associated with a decrease of several cardiovascular risk factors [8] and the presence of essential *n*-6 and *n*-3 FAs emphasise the importance of olives as an important food source of essential FAs. Besides FAs and phenolic compounds, the nutritional value of olives has also been associated with other lipids, like phytosterols, and non-lipid compounds, such as tocopherols and carotenoids [3].

In general, fruits are also sources of other lipids, as polar lipids. These lipids are structural components of the cellular membranes, are regulatory and signalling molecules, and they comprise several lipid classes, such as phospholipids and glycolipids. The amount of polar lipids has been estimated in the pulp of some lipid-rich fruits, as, for instance, avocado (*Persea americana* Mill.) fruit, with phospholipids accounting for 0.7–2.1% and glycolipids for 2.5–3.2% of the pulp’s lipid fraction [9], and the polar lipid molecular species were also reported [10]. Only a few papers reported the olive fruit’s polar lipid composition and have described the presence of several classes of phospholipids, glycolipids, and glycosphingolipids (reviewed by [11]). Around 0.12% phospholipids [12] and 0.028% glycolipids [13] were estimated. However, these studies relied on low-tech approaches, such as thin-layer chromatography (TLC), and the polar lipid profile of the olive fruit has not yet been comprehensively identified at the molecular level.

Some polar lipids have biological activity that is related to antioxidant capacity, memory, and play different roles in immunity and inflammation, and in the prevention of cardiovascular diseases [14]. Plant glycolipids have shown anti-inflammatory, anti-osteoporotic, and anti-tumour activity, both in vitro and in vivo [15,16]. The bioactive compounds responsible for these beneficial effects are far from being fully identified, even though several of these biological activities have been associated with the consumption of olives. Nevertheless, there are some studies on the polar lipid fraction of olive oil that showed in vitro and in vivo anti-thrombotic and anti-atherosclerotic activities. The olive oil’s polar lipid fraction inhibited platelet aggregation [17], and this effect was assigned to a glycerol glycolipid [18]. There is a growing interest in characterising and evaluating the positive impact of phospholipids, glycolipids, and glycosphingolipids in human health although the in vitro or in vivo biological activity of polar lipids from olives has never been tested. These studies aimed at discovering new bioactive compounds in olives and they will further support its claim as a functional food [11].

Due to the lack of knowledge in this field, this work aimed to describe the profile of TAGs and polar lipids of the olive pulp from Galega, a cultivar that is widely produced in Portugal, in detail. We have used a combined methodological approach relying on solid-phase extraction (SPE) for total lipid fractionation, analysis of TAGs by high-resolution C_30_ reversed-phase-liquid chromatography coupled to MS (RP-LC-MS), and analysis of polar lipids by high-resolution hydrophilic interaction liquid chromatography (HILIC)-normal phase-LC-MS and MS/MS to achieve this goal.

## 2. Results and Discussion

### 2.1. Triacylglycerol Profile

**Figure 1:**
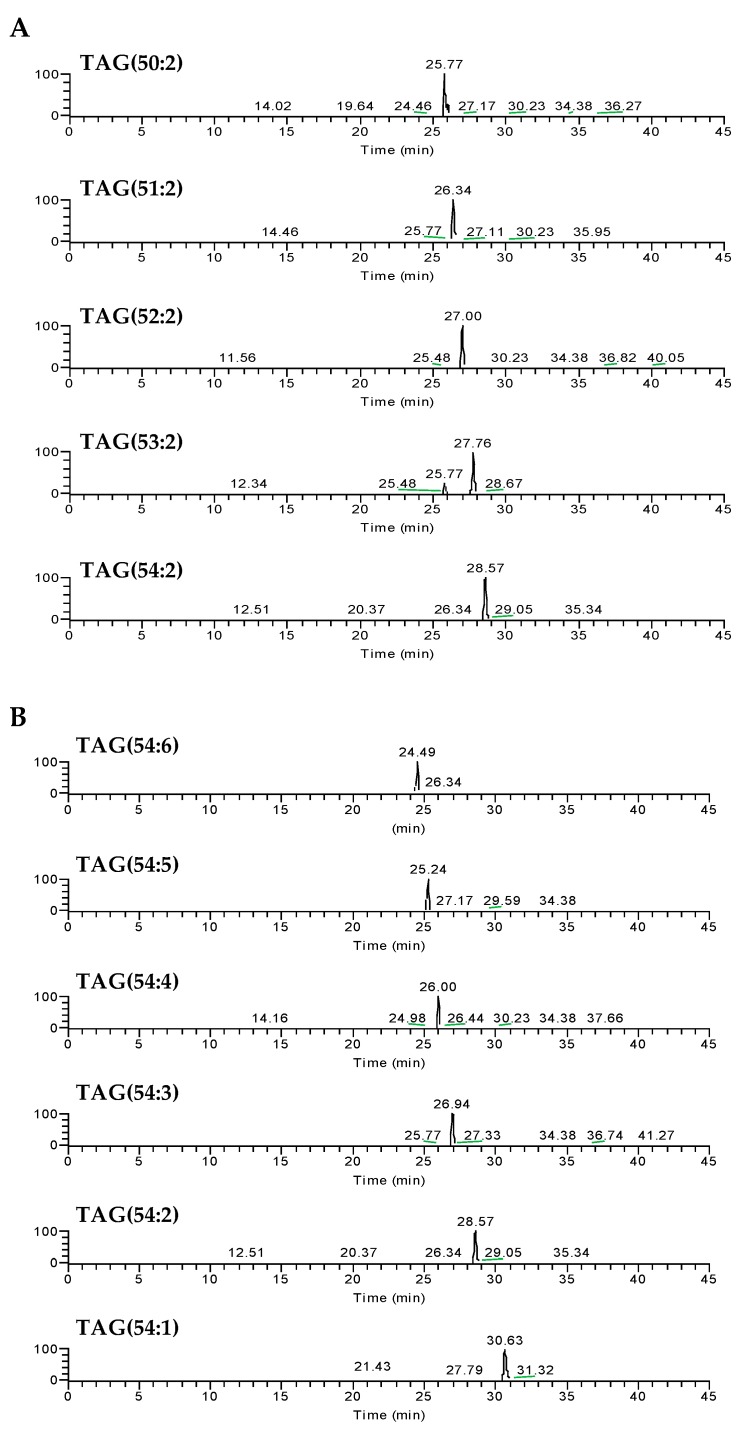
Representative reconstructed ion chromatograms (RIC) of several triacylglycerol (TAG) ions identified in the olive (*Olea europaea* L. cv. *Galega vulgar*) pulp, observed as [M + NH_4_]^+^ adducts. As an example, we show species with the same number of double bonds (2) and an increasing number of carbons (from 50 to 54) (**A**) and species with 54 carbons and decreasing number of double bonds (from 6 to 1) (**B**), obtained by C_30_ HPLC-ESI-MS.

The neutral lipid (NL)-rich fraction of the olive pulp that is mainly composed of TAGs was obtained by SPE of the total lipid extract, while using aminopropyl cartridges. This fraction was analysed by high-resolution reversed-phase C_30_ HPLC-ESI-MS/MS. In C_30_-LC chromatography, the retention time of the TAG molecular species increases as the number of carbon atoms increases (Figure 1A), as expected, and as the degree of unsaturation decreases (Figure 1B).

TAGs were observed in the LC-MS spectra, in the positive-ion mode, as [M + NH_4_]^+^ ions, and 71 different ions were assigned (Table 1). Subsequently, the TAG molecular species were identified by searching the measured exact mass against a custom database (error ≤ 5 ppm) and by the interpretation of the characteristic MS/MS fragmentation pattern of each ion. The analysis of MS/MS showed many isobaric species, and, while considering the different possible combinations of the fatty acyl chains that were identified by MS/MS analysis of each ion, a maximum of three hundred and seventy-three TAG compounds can be predicted in the olive pulp (Supplementary material, Table S1). Thirty one different fatty acyl chains that were esterified to TAGs were identified, which ranged from C11:0 to C26:0, including odd-chain FAs (11:0, 13:0, 15:0, 17:0, 19:0, 21:0, 23:0, and 25:0) and several PUFAs (16:2, 17:2, 18:2, 18:3, 18:4, 19:2, 20:2, and 20:3). The most abundant TAG species were identified in the LC-MS at *m/z* 902.81, *m/z* 876.80, *m/z* 874.78, and *m/z* 900.80, by descending order of abundance, corresponding to TAG 54:3, 52:2, 52:3, and 54:4, respectively. Each of these ions may correspond to more than one combination of the fatty acyl chains and Table S1 summarizes all of the TAG compounds that were identified by MS/MS (Supplementary material).

**Figure 2:**
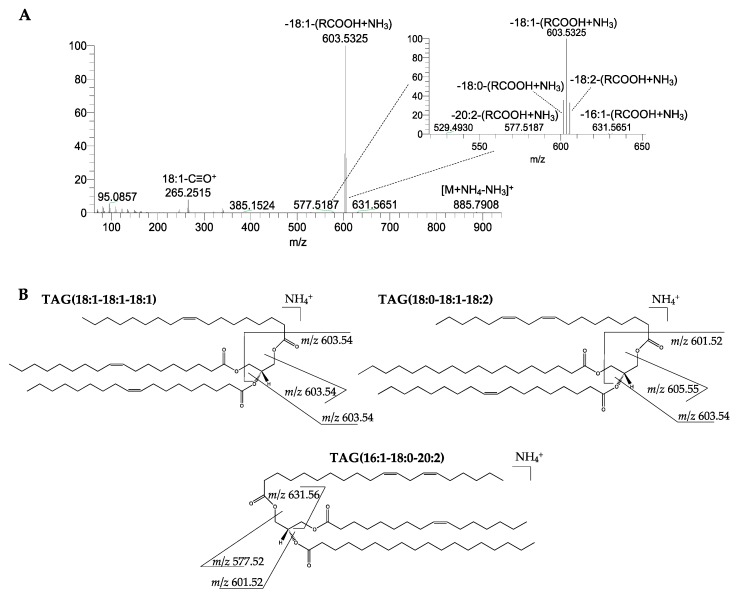
LC-MS/MS spectrum of the [M + NH_4_]^+^ ion at *m/z* 902.81 corresponding to TAG(54:3) identified in the olive (*Olea europaea* L. cv. *Galega vulgar*) pulp (**A**). The analysis of this MS/MS spectrum showed the neutral loss of RCOOH+NH_3_, suggesting the presence of six TAG molecular species: the most abundant as TAG(18:1-18:1-18:1), but also TAG(16:0-18:1-20:2), TAG(16:0-18:2-20:1), TAG(16:1-18:0-20:2), TAG(16:1-18:1-20:1), and TAG(18:0-18:1-18:2). The neutral loss of the acyl chains 16:0 and 20:1 are not observed in this spectrum. Proposed fragmentation pathways for TAG(18:1-18:1-18:1), TAG(16:1-18:0-20:2), and TAG(18:0-18:1-18:2) are shown (**B**).

The fatty acyl composition of the TAG molecular species was determined by manual analysis of the LC-MS/MS data. For example, the MS/MS spectra of TAG [M + NH_4_]^+^ ions showed a typical neutral loss of 17 Da, which corresponded to the neutral loss of ammonia (-NH_3_), and the neutral loss of each FA chain as a free carboxylic acid plus neutral ammonia (RCOOH+NH_3_), showing one or more diacylglycerol-like ions [19], depending on the composition of the fatty acyl chains (Figure 2). Acylium ions (RC≡O^+^) could also be seen in the *m/z* range between 200 and 300, which provides further information on the FA chains (Figure 2A).

TAGs are olives’ main lipid components, one of the most important groups of dietary lipids, and also the most studied. The characterisation of the TAG profile is relevant in understanding the quality characteristics and nutritional properties of olives. The physical properties of an oil are related to the type of FAs that are esterified to the glycerol backbone (MUFAs and PUFAs) and by the position of the FAs in the TAG molecule (*sn*-1, *sn*-2 and *sn*-3). This will affect the digestion, absorption, and metabolism of TAGs in the body [20,21].

In this work, the analysis of TAGs was achieved by using C_30_ in LC-MS, which is nowadays considered as one of the best approaches for the screening of this type of lipids [22]. C_30_ LC-MS has been used for TAG profiling in palm and canola oils, in which seventy molecular species were identified [23], and in biological specimens, such as rat plasma and liver [24]. The results from this study allowed for identifying seventy-one TAG molecular ions in the olive pulp’s NL-fraction that corresponded to more than three hundred fifty possible combinations of TAG molecular species. Previous studies reported nine to twelve TAGs on the pulp of different Italian olive varieties [7,25]; and eighteen TAGs in the Italian variety Coratina [5]. Herein, we have identified many TAG species with FAs that ranged from C11:0 to C26:0, several of which were different from that commonly reported for the olive pulp, such as 18:1, 16:0, 18:2*n*-6, 18:0, 16:1, and 18:3*n*-3 [5,7,25,26]. We identified TAGs with PUFAs other than 18:2 and 18:3, such as 16:2, 17:2, 19:2, 20:2, and 20:3. The most common TAGs found in olives are 50:1, 52:4, 52:2, 54:6, 54:5, 54:4, 54:3, and 54:2 [5,7,25,26], which are mainly composed of 18:1, 16:0, 18:2, and 18:0. Our results corroborate those from the literature and provide a much more complete TAG profile of the olive pulp that was unknown until now.

### 2.2. Polar Lipid Profile

**Figure 3:**
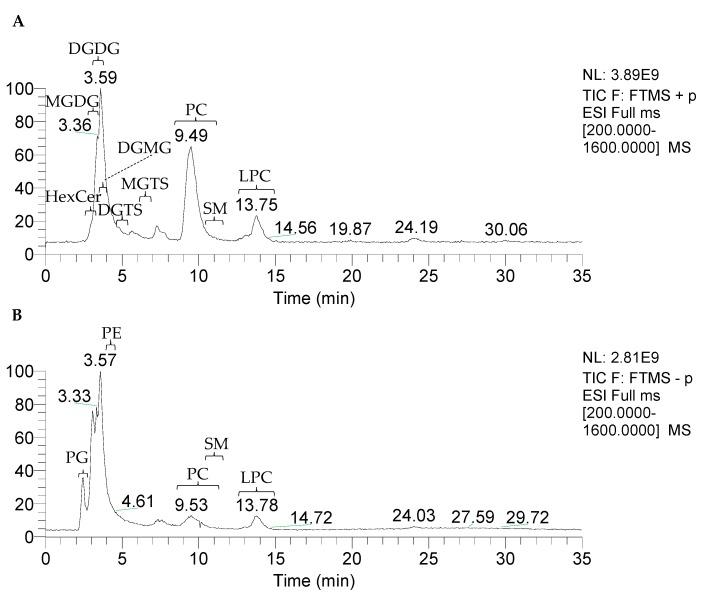
Total ion current (TIC) chromatograms of the polar lipid-rich fraction of the olive (*Olea europaea* L. cv. *Galega vulgar*) pulp in the positive-ion mode (**A**) and negative-ion mode (**B**) obtained by HILIC-ESI-MS. PG: phosphatidylglycerols; HexCer: hexosylceramides; MGDG: monoglycosyldiacylglycerols; DGDG: diglycosyldiacylglycerols; DGMG: diglycosylmonoacylglycerols; PE: phosphatidylethanolamines; DGTS: diacylglyceryl-*N*,*N*,*N*-trimethylhomoserines; MGTS: monoacylglyceryl-*N*,*N*,*N*-trimethylhomoserine; PC: phosphatidylcholines; SM: sphingomyelins; and, LPC: lyso-phosphatidylcholines.

The polar lipid-rich fraction of the olive pulp, as obtained by SPE of the total lipid extract, was analysed by high-resolution LC-MS/MS while using a HILIC column. As described for TAGs, the identification of polar lipids was based on the exact mass (error ≤ 5 ppm), the retention time, and analysis of MS/MS spectra of each ion. The total ion chromatogram in the positive-ion mode (Figure 3A) and in the negative-ion mode (Figure 3B) showed the elution of the lipid classes with different retention times, similarly as previously described [27,28]. Eleven classes of polar lipids were identified, including phospholipids, glycosphingolipids, glycolipids, and betaines (Figure 3). In total, one hundred and seven molecular species were identified: seventy-seven corresponding to phospholipids, nineteen to glycolipids, five to glycosphingolipids, and six to betaines (Table 2).

#### 2.2.1. Phospholipids

**Figure 4:**
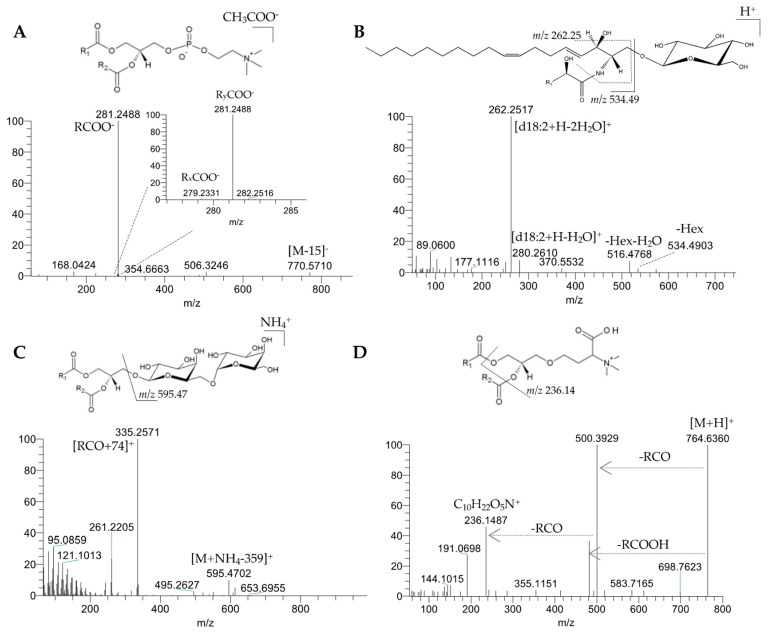
LC-MS/MS spectra of the different polar lipid classes identified in the olive (*Olea europaea* L. cv. *Galega vulgar*) pulp. One MS/MS spectrum illustrative of each group of polar lipids is shown: phospholipids—**A**) phosphatidylcholine PC(36:2) at *m/z* 844.60 as [M + CH_3_COO]^−^; glycosphingolipids—**B**) hexosylceramide HexCer(d34:2(OH)) at *m/z* 714.55 as [M + H]^+^; glyceroglycolipids—**C**) diglycosyldiacylglycerol DGDG(36:6) at *m/z* 954.61 as [M + NH_4_]^+^; and, betaines—**D**) diacylglyceryl-*N*,*N*,*N*-trimethylhomoserine DGTS(36:2) at *m/z* 764.63 as [M + H]^+^. A representative molecular structure of each polar lipid class is also shown.

Five classes of phospholipids were identified comprising seventy-seven molecular species: phosphatidylcholines (PC, forty-eight molecular species), lyso-phosphatidylcholines (LPC, twelve molecular species), sphingomyelins (SM, nine molecular species), phosphatidylethanolamines (PE, six molecular species), and phosphatidylglycerols (PG, two molecular species), as shown in Table 2. PC, LPC, SM, and PE were identified in the positive-ion mode, as [M + H]^+^ ions. In the negative-ion mode, PC, LPC, and SM were identified as [M + CH_3_COO]^−^ adducts, and PE and PG as [M − H]^−^ molecular ions. The identification by MS/MS was performed in the positive-ion mode to confirm the identity of the polar head groups, and in the negative-ion mode to confirm the fatty acyl composition [19]. The fatty acyl composition in all of the lipid species (except for SM) was corroborated by the presence of the carboxylate anions (RCOO^-^), which was also confirmed by the exact mass of these RCOO^-^ ions (Figure 4a for PC(36:2), as an example). As previously reported [19], in the MS/MS spectra of the [M + H]^+^ ions of PC, LPC, and SM, it was possible to identify the product ion at *m/z* 184.07, corresponding to the phosphocholine polar head group (C_5_H_15_NO_4_P^+^), which is typical of these lipid classes. MS/MS spectra of the [M + CH_3_COO]^−^ adducts of PC, LPC, and SM, showed a product ion at *m/z* 168.04, corresponding to the *N*-dimethylaminoethylphosphate anion. This product ion showed higher relative abundance in the MS/MS spectra of SM molecular species, together with the presence of another product ion at *m/z* 78.95, corresponding to HPO_3_^-^ [19].

PC molecular species with fatty acyl chains from 8:1 to 25:0 were identified, and most of the species contained the FA 18:1. The molecular species of PC bearing PUFAs 18:2 or 18:3 were identified, as well as other minor species with other PUFAs, such as 10:2, 16:2, 16:3, 17:2, 19:2, and 19:3. Long-chain hydroxy FA (monohydroxy and dihydroxy FAs) esterified to PC were detected (Table 2). Oxidised PC, corresponding to C9 short-chain oxidised products, one with an aldehyde terminal (C_8_CHO) and other with a carboxylic terminal (C_8_COOH), were also identified. LPC molecular species esterified to FAs ranging from C16 to C24 were observed, including hydroxylated LPC, as 18:1(OH), 18:2(OH), and 18:1(2OH) (Table 2). SM with dihydroxy and trihydroxy long-chain bases were identified: five molecular species with a dihydroxy long-chain base (“d” species) and four with a trihydroxy (phytosphingosine) long-chain base (“t” species). PE and PG were composed by FAs between C16 and C18, with up to four unsaturations, and no oxidised species being detected. The MS spectra showed that the most abundant species in each class were PC(36:2), LPC(18:1), SM(t38:1), PE(36:2), and PG(36:2). The fatty acyl chains of these molecules were mostly composed of 18:1.

Only one previous work characterized phospholipids in the olive pulp while using MS but using capillary electrophoresis as a separation method [29]. The authors disclosed seven classes in the pulp of Arbequina olives (PC, LPC, PE, PG, phosphatidic acid—PA, lyso-PA, and phosphatidylinositol—PI) and fifteen molecular species bearing 14:1, 16:0, 18:0, 18:1, 18:2, 18:3, 20:1, and 20:2 FAs. Only PG(34:1) and PG(36:2) were common to the present study, and no other polar lipid classes were detected. Other studies that were based on TLC stated that the olive pulp contains phospholipids [30], as cited by [29], glycolipids, sterol glucosides, cerebrosides, and sulfolipids [31], as cited by [32]. In the present study, many of these lipid classes were identified, and MS characterized their molecular species, providing a new insight on the polar lipid signature of the olive pulp. Several studies suggested that dietary phospholipids have antioxidant activity and they exert positive effects in cognitive function, immunity, inflammation, and hepatopathies, in preventing cardiovascular diseases, and several other conditions, especially PC and SM (reviewed by [14,33,34]). Food sphingolipids have demonstrated beneficial effects on atherosclerosis and colon cancer [35,36].

#### 2.2.2. Glycosphingolipids

Glycosphingolipids, namely hexosylceramides (HexCer), were identified in the polar lipid-rich fraction of the olive pulp. In the LC-MS, five molecular species of HexCer were identified as [M + H]^+^ ions and the most abundant ion was found at *m/z* 844.69, which corresponds to HexCer(t42:1(OH)) (Table 2). The MS/MS revealed both di- and tri-hydroxy long-chain bases (d18:1 or 8-sphingenine, d18:2 or 4,8-sphingadienine, and t18:1 or 4-hydroxy-8-sphingenine). The sphingoid base mainly contained the C18 amino alcohol, while the fatty acyl chains ranged between C16 and C26 and were hydroxylated. As an example, the MS/MS of HexCer(d34:2(OH)), found at *m/z* 714.55, is shown (Figure 4b). This MS/MS spectrum showed the neutral loss of a hexose (−180 Da, -Hex) and the combined loss of hexose plus H_2_O (−198 Da, -Hex-H_2_O) from the precursor ion. Likewise, product ions that were assigned to the sphingoid base were seen with high abundance at *m/z* 262.25 (loss of two water molecules from the long-chain base ([d18:2+H-2H_2_O]^+^) and, at lower abundance, at *m/z* 280.26 (loss of one water molecule from the long-chain base ([d18:2+H-H_2_O]^+^)) [37,38].

HexCer, which are also called cerebrosides, are glycosphingolipids that are found in cellular membranes and the tonoplast of plant cells. Earlier studies, utilizing TLC, revealed the presence of this lipid class in the olive pulp [30] cited by [29], but it has never been identified by LC-MS in the olive fruit. Some authors revealed the presence of HexCer in bell pepper and tomato, namely HexCer with d18:2, d18:1, and t18:1 sphingoid bases and C16:0 to C24:0 FAs, including hydroxy FAs [39]. In several nuts (e.g., almond) and seeds (e.g., pumpkin), HexCer identified by LC-MS/MS revealed both di-hydroxy and tri-hydroxy long-chain bases, mostly 4,8-sphingadienine (d18:2) [37]. The effective role of sphingolipid-derived metabolites on human colon cancer has been extensively studied in the last years [40]. Plant glycosphingolipids, such as soy glycosylceramide (GlcCer), suppressed colon tumorigenesis in mouse models [41], and sphingoid bases from wheat grain cerebrosides induced apoptosis in human colon cancer cells [42]. Dietary cerebrosides revealed other important physiological functions. HexCer were also associated with the reduction of plasma levels of cholesterol and TAGs, and with the prevention of liver steatosis in mice [43]. GlcCer from rice bran and germ improved the barrier function of the skin [44].

#### 2.2.3. Glycolipids

Three classes of glycolipids were identified in the olive pulp: monoglycosyldiacylglycerols (MGDG, nine molecular species), diglycosyldiacylglycerols (DGDG, eight molecular species), and diglycosylmonoacylglycerols (DGMG, two molecular species), as summarized in Table 2. These neutral glyceroglycolipids were detected in the positive-ion mode as [M + NH_4_]^+^ adducts. In the LC-MS, the most abundant MGDG and DGDG ions were found at *m/z* 792.56 and *m/z* 954.61, corresponding to MGDG(36:6) and DGDG(36:6), respectively. Glycolipids were both esterified to 18:3 FAs (Table 2). The MS/MS spectra of [M + NH_4_]^+^ ions of MGDG showed typical product ions resulting from the combined loss of NH_3_ (−17 Da) plus a hexose moiety (−180 Da), assigned as [M + NH_4_ − 197]^+^ [27]. Similarly, in the MS/MS spectra of the [M + NH_4_]^+^ ions of DGDG, it was possible to identify the product ions that were formed by the typical neutral loss of the DGDG headgroup, corresponding to two hexoses (loss of 162 Da + 180 Da) combined with the loss of NH_3_ (−17 Da), as [M + NH_4_ − 359]^+^. Characteristic acylium ions plus 74 ([RCO + 74]^+^), which were formed by combined loss of one FA and the hexose moiety, allowed for the identification of the fatty acyl composition of MGDG and DGDG molecular species [27,45]. As an example, Figure 4c depicts the MS/MS of DGDG(36:6). The two molecular species of DGMG, DGMG(18:1) and DGMG(18:3), were only identified by exact mass and retention time, since no informative MS/MS spectra could be obtained.

The MGDG glycolipids that were identified herein were structurally characterised for the first time in the olive pulp. DGDG were also found, which is in accordance with a previous report by Bianco et al. (1998) that identified two species of DGDG (18:3-18:3 and 18:1-18:3) in the olive pulp while using HPLC-UV [13]. The isolated glycolipids or glycolipid-rich fractions, including MGDG and DGDG, have shown to possess in vitro and in vivo biological activity. MGDG that was isolated from spinach (*Spinacia oleracea* L.) inhibited the growth of human gastric cancer cells [46] and pancreatic cancer cells combined with X-ray radiation [47]. DGDG(18:3-18:3) that was isolated from dog rose (*Rosa canina* L.) revealed anti-inflammatory activity [48]. Plant and fruit glycolipids, similarly to algae glycolipids, are phytochemicals with potential health benefits, despite being scarcely explored for their biological activity (reviewed by [49]).

#### 2.2.4. Betaine Lipids

In this work, we have identified betaine lipids in the diacyl and monoacyl forms (five diacylglyceryl-*N*,*N*,*N*-trimethylhomoserines (DGTS) and one monoacylglyceryl-*N*,*N*,*N*-trimethylhomoserine (MGTS)). DGTS and MGTS were identified as [M + H]^+^ ions and the MS/MS showed a typical product ion of these classes, at *m/z* 236.14, which corresponded to the ion C_10_H_22_O_5_N^+^ generated from the cleavage of the ester bonds and the loss of both acyl chains [50]. The neutral loss of each acyl chain as ketene (-RCO) and as an acid (-RCOOH) from the precursor ion was also seen. The most abundant DGTS was assigned as DGTS(36:2), being composed of two 18:1 acyl chains. Figure 4d depicts the MS/MS of DGTS(36:2), as an example.

Betaine lipids are complex, naturally occurring, extra-plastidial lipids, which contain a betaine moiety as a polar head group linked to the *sn*-3 position of glycerol by an ether bond, having one or two esterified FAs. Betaine lipids, such as DGTS, are commonly found in protozoa, fungi, photosynthetic bacteria, algae, and lower plants as bryophytes [51], but they have not been reported for higher plants. However, DGTS(34:1) has been recently identified in virgin olive oil [52], corroborating the results from the present study. These lipids are acknowledged to completely replace phospholipids in membranes of many algae and fungi under stress conditions, as phosphate deprivation. The biological role of betaine lipids is still unknown. However, recent research has revealed that betaine supplementation promotes lipid metabolism, improves insulin resistance in obese mice [53], lowers the expression of inflammatory markers [54], and has favourable effects on liver function-related metabolism in humans [55].

The results of the relative quantification of polar lipids (Table 3) showed that phospholipids (71.96%) were the most abundant lipid class, followed by glycolipids (25.44%), glycosphingolipids (2.33%), and betaines (0.54%). Within the phospholipid classes, PC was the most abundant class (96.66%). The relative abundances of glycolipids were equally distributed between MGDG (52.29%) and DGDG (46.97%).

The PC hydroxylated molecular species represented 14.58% of the total PC and LPC hydroxylated molecular species corresponded to 2.89% of total LPC (Table 3). All of the HexCer molecular species also found herein bear hydroxylated acyl chains. Hydroxylated FAs (oxylipins) are bioactive metabolites originating from the oxidation of unsaturated FAs, such as 18:1, 18:2, 18:3, etc., under the complex action of lipoxygenases. These unsaturated hydroxylated FAs, mainly 18:3(OH), 18:2(OH), 18:1(OH), 18:1(2OH), and 18:2(2OH), have been identified in higher plants [56,57,58,59]. Hydroxyoctanoic acid (8:0(OH)) and other short-chain hydroxy FAs were identified in the soil system that was associated with *Calluna* plants [60]. Medium-chain, saturated, monounsaturated, and polyunsaturated oxidized FAs, such as 8:0(OH), 8:1(OH), 14:0(OH), 14:1(OH), 16:2(OH), and 16:3(OH), have been reported in *Arabidopsis thaliana* [61]. The role of dietary oxylipins derived from *n*-3 and *n*-6 PUFAs in maintaining health and preventing disease is still uncertain or controversial, but some studies pointed out the beneficial health effects (reviewed by [62,63]).

In this work, several FAs that are not commonly detected in olives were identified. Based on LC-MS/MS data analysis, odd and polyunsaturated FAs were detected on TAGs. Additionally, we have identified odd, polyunsaturated, hydroxylated, and short-chain FAs on polar lipids. To illustrate these findings, the LC-MS/MS spectra of TAG(53:3), TAG(48:3), PC(36:3(OH)), and PC(26:2) are provided (Supplementary material, Figures S2 and S3).

Over the past century, many studies on higher plants and plant seed oils extensively detected the unusual FAs (reviewed by [56]). Unusual odd FAs (saturated and unsaturated) are known to be present in plants, bacteria, filamentous fungi, yeasts, algae, and protozoa (reviewed by [64]). Odd FAs as 15:1, 17:1, 19:0, 23:0, and 25:0 and polyunsaturated FAs (16:2, 17:2, and 18:4) were identified in alpine plants [65]. More recently, several of these FAs were identified in microalgae that were esterified to the polar lipids [66]. Unusual FAs have also been observed in TAGs [67]. It is known that plants tolerate high levels of unusual FAs in storage lipids, because they are retained in lipid droplets and have no structural function. On the other hand, PCs are the substrate for the synthesis of unusual FAs, such as hydroxy ones [68]. In addition, studies in seed oils showed that some of these unusual FAs are produced from PC in the endoplasmic reticulum and then selectively accumulate in the TAGs [67]. These FAs are not commonly found in olives, as they are trace compounds. However, the sensitivity and resolution of the analytical approach used herein has allowed their identification. Nevertheless, the study of these metabolites goes beyond the scope of this work and it will have to be investigated in further studies to better understand the nutritional properties of olives and olive oil and how they affect their quality.

## 3. Materials and Methods 

### 3.1. Reagents

All of the organic solvents used had HPLC purity and they were purchased from Fisher Scientific Ltd. (Loughborough, UK): chloroform (CHCl_3_), methanol (MeOH), *n*-hexane, acetonitrile, and diethyl ether. Other reagents were purchased from major commercial sources. Purified water (Synergy, Millipore Corporation, Billerica, MA, USA) was used whenever necessary. Phospholipid internal standards were obtained from Avanti Polar Lipids, Inc. (Alabaster, AL; USA): 1,2-dimyristoyl-*sn*-glycero-3-phosphocholine (dMPC); 1-nonadecanoyl-2-hydroxy-*sn*-glycero-3-phosphocholine (LPC); *N*-palmitoyl-d-*erythro*-sphingosylphosphorylcholine (NPSM); 1,2-dimyristoyl-*sn*-glycero-3-phosphoethanolamine (dMPE); 1,2-dimyristoyl-*sn*-glycero-3-phospho-(1′-rac-glycerol) (dMPG); 1,2-dimyristoyl-*sn*-glycero-3-phospho-l-serine (dMPS); 1,2-dipalmitoyl-*sn*-glycero-3-phosphatidylinositol (dPPI); 1,2-dimyristoyl-*sn*-glycero-3-phosphate (dMPA); 1,1′,2,2′-tetramyristoyl cardiolipin; and, *N*-heptadecanoyl-d-*erythro*-sphingosine (C17 Cer(d35:1)).

### 3.2. Sampling and Sample Preparation

Galega olive samples were randomly collected in the 2016/2017 campaign, in December 2016, in a traditional olive orchard from the Nelas region (Nelas, Portugal) and then transported to the laboratory at 4 °C. In the laboratory, the olives were washed with tap water, then with distilled water, and then dried with a cloth. Degraded or diseased olives were discarded. The pulp was removed with a knife and immediately frozen in liquid nitrogen. Subsequently, it was transferred to screw cap vials, lyophilised, and stored away from light and moisture until lipid extraction.

### 3.3. Total Lipid Extraction

The Bligh and Dyer method [69] was used for extracting the total lipids from the olive pulp. Three hundred mg of lyophilised pulp were placed in an iced glass tube, 1 mL of ultrapure water was added, and the mixture was completely homogenised (Diax 900 Heidolph, Sigma-Aldrich, Darmstadt, Germany) in a few seconds. Afterwards, 3.75 mL of CHCl_3_/MeOH (1:2, by volume) was added, homogenised, and the samples were left on ice 30 min. Afterwards, 1.25 mL of CHCl_3_ and 1.25 mL of water were added, homogenised in between, and centrifuged for 10 min. at 142 ×*g*. The lipids (organic phase, bottom layer) were transferred to a new glass tube. Finally, 1.88 mL of CHCl_3_ were added to the aqueous phase, and the mixture was centrifuged again. The organic phase was added to the first organic phase and then dried under a nitrogen stream. After concentration in nitrogen and redissolution in CHCl_3_, the lipids were transferred to glass amber vials. The dried extracts were stored at −20 °C until fractionation. Lipid extraction was performed five times (*n* = 5) on the same day.

### 3.4. Total Lipid Fractionation

Total lipid extracts were fractionated using aminopropyl SPE cartridges (Discovery DSC-NH_2_, ref. 52637-U Supelco, Sigma-Aldrich), coupled to a vacuum manifold (Visiprep SPE Vacuum Manifold, ref. Supelco-57030-U, Sigma-Aldrich) to obtain lipid fractions of different lipid classes (NL, intermediate polarity lipids and polar lipids). The procedure was adapted from Ruíz et al. (2004) [70]. Briefly, it consisted of conditioning the cartridge with *n*-hexane; loading with the lipid extract (40 mg dissolved in CHCl_3_); elution of the NL with CHCl_3_ (fraction 1); elution of lipids of intermediate polarity with diethyl ether/acetic acid (98:2, by volume, fraction 2); and, finally, the elution of the polar lipids with CHCl_3_/MeOH (1:6, by volume, fraction 3) and CHCl_3_/MeOH (1:1, by volume, fraction 4). Each fraction was collected into glass tubes, dried under nitrogen and transferred to 2 mL amber glass vials. The yield of the NL fraction was determined by gravimetry. The amount of phospholipids was determined before HPLC-MS analysis in the polar lipid-rich fractions (fractions 3 and 4 were combined), according to Rouser et al. (1970) [71].

### 3.5. HPLC-MS and HPLC-MS/MS Analysis of the Neutral Lipid Fraction

The NL-rich fraction that was obtained after SPE (fraction 1) was analysed by HPLC-ESI-MS and HPLC-ESI-MS/MS while using a reversed phase column with a C_30_ stationary phase. The UltiMate 3000™ UHPLC system (Thermo Fisher Scientific, Germering, Germany) was coupled online to a Q Exactive™ HF hybrid quadrupole-Orbitrap mass spectrometer (Thermo Fisher, Scientific, Bremen, Germany). The mobile phases consisted of water/acetonitrile (50:50, by volume) with 0.1% formic acid and 5 mM ammonium formate (phase A), and isopropanol/acetonitrile/water (85:10:5, by volume) with 0.1% formic acid and 5 mM ammonium formate (phase B). The solvent gradient was set up with an initial ramp from 50% B to 86% B at 20 min., followed by an increase to 95% B in 1 min. (at 21 min), which was isocratically held for 14 min., followed by a decrease to 50% in 2 min. (at minute 37), and maintained for 8 min. until the end of the run (at 45 min). The flow rate was 300 µL min^−1^. The lipid fraction was diluted to the final concentration of 200 ng·µL^−1^ in MeOH, and 2 µL (corresponding to 0.4 µg) of the diluted mixture were introduced into an Accucore™ C_30_ column (150 × 2.1 mm) that was equipped with 2.6 µm diameter fused-core particles (Thermo Fisher Scientific, Germering, Germany).

The Q Exactive™ HF hybrid quadrupole-Orbitrap mass spectrometer operated in positive-ion mode (electrospray voltage 3 kV) on a mass range comprised between 400 and 1600 *m/z*, with a resolution of 70,000, a maximum injection time of 100 ms, and an AGC target of 1 × 10^6^ throughout the full MS experiments. The capillary temperature was 350 °C; sheath gas and the auxiliary gas flows were 45 arbitrary units (AU) and 15 AU, respectively.

High collision dissociation (HCD)-tandem mass spectra of [M + NH_4_]^+^ precursor ions were obtained with a resolution of 17,500, a maximum injection time of 200 ms, an automatic gain control (AGC) target of 2 × 10^3^ and with cycles consisting of one MS survey, followed by ten data-dependent MS/MS events, with an isolation window of 1 *m/z*, a dynamic exclusion of 60 s, and an intensity threshold of 5 × 10^4^. Normalized collision energy™ (NCE) was stepped between 20, 23, and 25 eV. Five injections (*n* = 5) were performed for the pulp’s NL-rich fractions for the full scan MS run and MS/MS experiments.

### 3.6. HPLC-MS and HPLC-MS/MS Analysis of the Polar Lipid Fraction 

The polar lipid-rich fraction obtained after SPE (fractions 3 and 4 were combined) was analysed by HPLC-ESI-MS and HPLC-ESI-MS/MS while using a HILIC column, on the same HPLC system that was mentioned in Section 3.5, which was coupled online to the Q-Exactive Orbitrap mass spectrometer. The solvent system consisted of two mobile phases (eluent A and eluent B). Eluent A consisted of acetonitrile/MeOH/water, 50:25:25, by volume, with 1 mM ammonium acetate. Eluent B consisted of acetonitrile/MeOH, 60:40, by volume, with 1 mM ammonium acetate. Initially, 0% of mobile phase A was held isocratically for 8 min., followed by a linear increase to 60% of A within 7 min. and a maintenance period of 15 min., returning to the initial conditions in 10 min. A volume of 5 μL of each sample, containing 5 μg of lipid fraction dissolved in CHCl_3_, 4 μL of a mix of phospholipid standards (dMPC, 0.02 μg; dMPE, 0.02 μg; NPSM, 0.02 μg; LPC, 0.02 μg; dPPI, 0.08 μg; dMPG, 0.012 μg; dMPS, 0.04 μg; dMPA, 0.08 μg; C17 Cer(d35:1), 0.02 μg), and 91 μL of eluent B, was introduced into the Ascentis Si column HPLC Pore column (15 cm × 1 mm, 3 μm, Sigma-Aldrich), with a flow rate of 40 μL·min^−^^1^, at 30 °C. Five injections were performed for the pulp’s polar lipid-rich fractions for the full scan MS run (*n* = 5). A mix of the five samples was used for the MS/MS experiments.

The mass spectrometer was simultaneously operated in positive (electrospray voltage was 3.0 kV) and negative (electrospray voltage was −2.7 kV) ionisation modes, with a high resolution (70,000) and AGC target of 1 × 10^6^. The capillary temperature was 250 °C and the sheath gas flow was 15 AU. In the MS/MS experiments, a resolution of 17,500 and AGC target of 1 × 10^5^ were used. The cycles consisted of one full scan mass spectrum and ten data-dependent MS/MS scans that were continuously repeated throughout the experiments with the dynamic exclusion of 60 s and intensity threshold of 1 × 10^4^, NCE ranged between 25, 30, and 35 eV.

### 3.7. Data Analysis

Data acquisition was carried out while using the Xcalibur data system (V3.3, Thermo Fisher Scientific, Waltham, MA, USA). The identification of TAG and polar lipid molecular species was based on the assignment of the precursor ions that were observed in LC–MS spectra and by the identification of the well-known fragmentation pattern of each class observed in the MS/MS spectra of each ion, as described in the literature, expected retention time, and mass accuracy with an error of ≤ 5 ppm.

The raw data were processed while using the MZmine software version 2.32 [72]. First, the mass list was filtered, followed by peak detection and peak processing. During the processing of the raw data, acquired in full MS mode, only peaks with raw intensity upper than 1 × 10^4^ and with a mass tolerance of ≤ 5 ppm were considered. Peak assignment and ion identification that are based on mass accuracy were performed against in-house databases for TAGs and polar lipids, built up based on the LIPID MAPS^®^ database.

The relative quantification of the polar lipids was calculated for the polar lipid families (phospholipids, glycolipids, glycosphingolipids, and betaines), the polar lipid classes within each family (PC, LPC, PE, SM, PG, MGDG, DGDG, DGMG, HexCer, DGTS, MGTS), and for the non-hydroxylated and hydroxylated molecular species within the PC and LPC classes. It was performed while using the chromatographic peak area values of each ion. Data normalization was performed by dividing the areas of the ions corresponding to the molecular species of each class by the area of the ions that were assigned as the internal standard of each class [72]. Since no internal standard was used for the HexCer, MGDG, DGDG, and DGMG classes, they were normalized while using the ceramide internal standard (Cer(d18:1-17:0)), which had the same retention time. Additionally, the DGTS and MGTS classes were normalized while using the PE standard (PE(14:0-14:0)) for the same reason.

## 4. Conclusions and Future Perspective

Olives are primordial sources of plant-derived lipids in the Mediterranean diet. Thorough knowledge of the olive’s lipidome is needed to evaluate its nutritional value and the health benefits that are assigned to this diet. In this work, a detailed characterisation of the triacylglycerol and polar lipid profiles of the olive pulp was achieved with liquid-chromatography/high-resolution mass spectrometry-based lipidomics. It revealed an updated list of triacylglycerols with more than three hundred and fifty molecules, bearing saturated, monounsaturated, polyunsaturated, even, odd, medium-chain, and long-chain fatty acids. The polar lipidome includes more than a hundred molecules from distinct classes and sub-classes of lipids, comprising phospholipids, glycolipids, glycosphingolipids, and betaines, and bearing saturated, monounsaturated, polyunsaturated (including essential fatty acids 18:2*n*-6 and 18:3*n*-3), monohydroxy, and dihydroxy fatty acids.

The use of both C_30_-LC- and HILIC-LC-ESI-MS and MS/MS allowed for the identification of a wide variety of lipid species, some with putative biological activities, which will bring new insight into their role in the health benefits of olives as functional ingredients of the Mediterranean diet. This approach also allows for better knowledge of the lipid composition of the olive pulp. This may be important to obtain deeper insight into the nutritional aspects of the currently commercialized products (olive flesh and virgin olive oil) and to design new products (virgin olive oils only obtained from the pulp, dehydrated olives, among others) with an optimized composition that is based on their bioactive lipid components. Moreover, this lipidomic phenotyping may be important for the bioprospection and valorisation of table olives on the food market. 

This study demonstrated that lipidomics of the neutral lipid and polar lipid fractions of the olive pulp is a key tool in fingerprinting the major and minor lipid profiles and to discover new compounds with potential biological activity. Therefore, these compounds can be exploited at different levels in the food industry and, beyond, in different market segments that use olives as an ingredient or raw material, such as in the nutraceutical, dermocosmetic, pharmaceutical, and animal feed industries.

## Figures and Tables

**Table 1:** Molecular species of triacylglycerols (TAGs) identified in the olive (*Olea europaea* L. cv. *Galega vulgar*) pulp by C_30_-reversed-phase-high-performance liquid chromatography-electrospray ionization-orbitrap-mass spectrometry (RP-HPLC-ESI-orbitrap-MS) and MS/MS analysis, with the identification of the major isobaric species.

Lipid name	*m/z*	*t*_R_ (min)	Acyl Chain Composition
TAG(C:N)	[M + NH_4_]^+^		
TAG(40:0)	712.6446	23.02	12:0-12:0-16:0 and 12:0-14:0-14:0
TAG(41:0)	726.6595	23.29	13:0-14:0-14:0
TAG(42:1)	738.6597	23.02	12:0-12:0-18:1 and 12:0-14:0-16:1
TAG(42:0)	740.6758	23.78	12:0-14:0-16:0 and 13:0-14:0-15:0
TAG(43:1)	752.6757	23.39	13:0-14:0-16:1
TAG(43:0)	754.6914	23.95	14:0-14:0-15:0
TAG(44:2)	764.6763	23.18	14:0-14:1-16:1
TAG(44:1)	766.6916	23.88	14:0-14:0-16:1
TAG(44:0)	768.7071	24.67	14:0-15:0-15:0
TAG(45:2)	778.6913	23.56	14:1-15:0-16:1
TAG(45:1)	780.7065	24.33	14:0-15:0-16:1
TAG(45:0)	782.7230	25.20	14:0-15:0-16:0
TAG(46:3)	790.6922	23.32	14:1-16:1-16:1
TAG(46:2)	792.7069	23.98	15:0-15:1-16:1
TAG(46:1)	794.7226	24.84	14:0-16:0-16:1 and 14:1-16:0-16:0
TAG(46:0)	796.7386	25.77	14:0-16:0-16:0 and 15:0-15:0-16:0
TAG(47:3)*	804.7074	23.71	47:3
TAG(47:2)	806.7227	24.46	15:0-16:1-16:1
TAG(47:1)	808.7380	25.31	15:0-16:0-16:1
TAG(47:0)	810.7541	26.38	15:0-16:0-16:0
TAG(48:3)	818.7231	24.09	14:1-16:1-18:1
TAG(48:2)	820.7386	24.98	16:0-16:1-16:1
TAG(48:1)	822.7544	25.77	16:0-16:0-16:1
TAG(48:0)	824.7700	27.07	16:0-16:0-16:0
TAG(49:3)	832.7384	24.52	16:1-16:1-17:1
TAG(49:2)	834.7539	25.39	15:1-16:0-18:1
TAG(49:1)	836.7698	26.38	15:0-16:0-18:1
TAG(49:0)	838.7851	27.85	16:0-16:0-17:0
TAG(50:4)	844.7388	24.33	16:1-16:1-18:2
TAG(50:3)	846.7541	24.98	16:0-16:1-18:2
TAG(50:2)	848.7708	25.77	16:0-16:1-18:1
TAG(50:1)	850.7866	27.04	16:0-16:0-18:1
TAG(50:0)	852.8004	28.70	16:0-16:0-18:0
TAG(51:3)	860.7699	25.48	16:0-17:1-18:2 and 16:1-17:0-18:2 and 16:1-17:1-18:1
TAG(51:2)	862.7856	26.34	16:0-17:0-18:2 and 16:0-17:1-18:1
TAG(51:1)	864.8012	27.76	16:0-17:0-18:1
TAG(51:0)	866.8158	29.72	16:0-17:0-18:0
TAG(52:4)	872.7700	25.27	16:0-18:2-18:2
TAG(52:3)	874.7866	25.77	16:1-18:1-18:1
TAG(52:2)	876.8027	27.00	16:0-18:1-18:1
TAG(52:1)	878.8169	28.63	16:0-18:0-18:1
TAG(52:0)	880.8319	30.75	16:0-18:0-18:0
TAG(53:6)*	882.7509	25.77	53:6
TAG(53:4)	886.7861	25.42	17:1-18:1-18:2
TAG(53:3)	888.8013	26.34	17:1-18:1-18:1
TAG(53:2)	890.8167	27.76	17:0-18:1-18:1
TAG(53:1)	892.8316	27.11	17:0-18:0-18:1
TAG(54:6)	896.7697	24.49	18:2-18:2-18:2
TAG(54:5)	898.7859	25.24	18:1-18:1-18:3
TAG(54:4)	900.8021	26.00	18:1-18:1-18:2
TAG(54:3)	902.8182	26.94	18:1-18:1-18:1
TAG(54:2)	904.8333	28.57	18:0-18:1-18:1
TAG(54:1)	906.8480	30.63	18:0-18:0-18:1
TAG(54:0)	908.8640	33.39	14:0-16:0-24:0
TAG(55:4)	914.8184	25.27	18:1-18:2-19:1
TAG(55:2)	918.8482	27.04	18:1-18:1-19:0
TAG(55:1)	920.8642	28.63	16:0-18:1-21:0
TAG(56:5)	926.8155	26.00	18:1-18:3-20:1
TAG(56:4)	928.8332	27.00	18:1-18:1-20:2
TAG(56:3)	930.8482	28.41	18:1-18:1-20:1
TAG(56:2)	932.8640	30.59	18:1-18:1-20:0
TAG(56:1)	934.8802	22.45	16:0-18:1-22:0 and 18:0-18:1-20:0
TAG(57:5)*	940.8334	25.24	57:5
TAG(57:4)*	942.8490	26.00	57:4
TAG(57:3)*	944.8640	26.91	57:3
TAG(57:2)	946.8792	28.57	18:1-18:1-21:0
TAG(58:3)	958.8793	31.25	18:1-18:2-22:0
TAG(58:2)	960.8956	33.19	18:1-18:1-22:0
TAG(58:1)	962.9106	23.08	16:0-18:1-24:0
TAG(60:3)	986.9110	33.98	18:1-18:2-24:0
TAG(60:2)	988.9265	36.51	18:1-18:1-24:0

All molecular species were identified by the analysis of the MS/MS spectrum, exact mass and retention time, except the molecular species with an asterisk (*) that were identified only by exact mass and by retention time since no MS/MS spectrum was available. C:N represents the total number of carbons (C) and the number of double bonds (N). *t*_R_, retention time. The fatty acyl chains of the indicated TAG molecular species were assigned based on the major FA fragment ions observed in the MS/MS spectra of each TAG. Detailed information on the TAG molecular species is listed in Table S1 of Supplementary material with the identification of all possible isobaric composition based on the other fragments found in the MS/MS of each TAG.

**Table 2:** Molecular species of polar lipids (phospholipids, glycolipids, glycosphingolipids and betaines) identified in the olive (*Olea europaea* L. cv. *Galega vulgar*) pulp by HILIC-LC-ESI-MS and MS/MS.

Lipid Name	*m/z*	*t*_R_ (min)	Acyl Chain Composition
Class(C:N)	[M + H]^+^		
PC(24:0(OH))	638.4399	11.93	16:0-8:0(OH)
PC(26:2)	646.4436	10.13	8:1-18:1
PC(16:0/C8CHO)*	650.4400	10.93	16:0/C8CHO
PC(26:2(OH))	662.4403	10.83	18:1-8:1(OH)
PC(26:1(OH))	664.4585	11.63	18:1-8:0(OH)
PC(16:0/C8COOH)*	666.4348	10.83	16:0/C8COOH
PC(27:2(OH))	676.4560	10.63	18:1-9:1(OH)
PC(28:3(OH))	688.4554	10.53	18:1-10:2(OH)
PC(30:3)^a)^	700.4895	10.03	30:3
PC(29:2(OH))	704.4867	10.43	18:1-11:1(OH)
PC(32:2)	730.5379	9.63	16:1-16:1
PC(32:1)	732.5539	9.63	16:0-16:1 and 14:0-18:1
PC(32:2(OH))	746.5316	9.83	16:0-16:2(OH) and 16:1-16:1(OH) and 18:1-14:1(OH)
PC(33:1)	746.5671	9.53	16:0-17:1
PC(32:1(OH))	748.5476	9.83	18:1-14:0(OH) and 16:0-16:1(OH)
PC(34:4)	754.5372	9.53	16:1-18:3
PC(34:3)	756.5545	9.43	16:0-18:3 and 16:1-18:2
PC(34:2)	758.5699	9.43	16:0-18:2 and 16:1-18:1
PC(34:1)	760.5854	9.43	16:0-18:1
PC(34:4(OH))	770.5338	9.73	17:1-17:3(OH) and 18:1-16:3(OH) and 18:2-16:2(OH)
PC(35:3)	770.5697	9.43	18:2-17:1
PC(35:2)	772.5848	9.23	18:1-17:1
PC(34:3(OH))	772.5487	9.63	16:0-18:3(OH) and 16:1-18:2(OH) and 18:1-16:2(OH)
PC(34:2(OH))	774.5646	9.63	16:1-18:1(OH) and 16:0-18:2(OH) and 18:1-16:1(OH)
PC(35:1)	774.5998	9.23	18:1-17:0
PC(34:1(OH))	776.5799	9.63	16:0-18:1(OH)
PC(36:6)	778.5380	9.43	18:3-18:3
PC(36:5)	780.5512	9.43	18:3-18:2
PC(36:4)	782.5689	9.33	18:2-18:2 and 18:3-18:1
PC(36:3)	784.5853	9.23	18:1-18:2
PC(36:2)	786.6009	9.03	18:1-18:1
PC(34:1(2OH))	792.5756	8.83	16:0-18:1(2OH)
PC(36:5(OH))	796.5476	9.63	18:2-18:3(OH)
PC(37:4)	796.5829	9.13	18:1-19:3 and 18:2-19:2
PC(36:4(OH))	798.5647	9.43	18:1-18:3(OH) and 18:2-18:2(OH)
PC(36:3(OH))	800.5798	9.33	18:1-18:2(OH) and 18:2-18:1(OH)
PC(37:2)	800.6156	9.03	19:0-18:2 and 18:1-19:1
PC(36:2(OH))	802.5949	9.43	18:1-18:1(OH)
PC(38:3)	812.6140	8.93	18:1-20:2 and 18:2-20:1
PC(38:2)	814.6318	8.93	18:1-20:1
PC(38:1)	816.6452	8.83	18:1-20:0
PC(36:3(2OH))	816.5755	8.73	18:1-18:2(2OH)
PC(36:2(2OH))	818.5908	8.73	18:1-18:1(2OH)
PC(40:2)	842.6611	8.83	18:1-22:1 and 18:2-22:0
PC(40:1)	844.6812	8.73	18:1-22:0
PC(41:1)	858.6943	8.66	18:1-23:0
PC(42:1)	872.7111	8.63	18:1-24:0
PC(43:1)	886.7261	8.53	18:1-25:0
	**[M + H]^+^**		
LPC(16:1)	494.3245	14.03	16:1
LPC(16:0)	496.3402	13.73	16:0
LPC(18:3)	518.3224	13.73	18:3
LPC(18:2)	520.3404	13.63	18:2
LPC(18:1)	522.3559	13.43	18:1
LPC(18:2(OH))	536.3355	14.53	18:2(OH)
LPC(18:1(OH))	538.3511	14.63	18:1(OH)
LPC(20:1)	550.3876	12.93	20:1
LPC(20:0)	552.4025	12.73	20:0
LPC(18:1(2OH))	554.3457	13.13	18:1(2OH)
LPC(22:0)	580.4359	12.42	22:0
LPC(24:0)	608.4664	11.93	24:0
	**[M + H]^+^**		
PE(30:3)^a)^	658.4426	4.54	
PE(34:2)	716.5216	4.34	16:1-18:1 and 16:0-18:2
PE(34:1)	718.5380	4.34	16:0-18:1
PE(36:4)^*^	740.5216	4.34	
PE(36:3)	742.5388	4.34	18:1-18:2
PE(36:2)	744.5538	4.24	18:1-18:1 and 18:0-18:2 and 17:1-19:1
	**[M − H]^−^**		
PG(34:1)	747.5175	2.49	16:0-18:1
PG(36:2)^*^	773.5332	2.49	
	**[M + H]^+^**		
SM(d34:1)	703.5755	11.33	
SM(d36:1)	731.6054	11.13	
SM(t38:1)*	775.6330	10.63	
SM(d41:1)	801.6875	10.53	
SM(t40:0)	805.6792	10.83	
SM(d42:2)	813.6870	10.53	
SM(d42:1)	815.6969	10.53	
SM(t41:1)*	817.6834	10.33	
SM(t41:0)	819.6953	10.73	
	**[M + NH_4_]^+^**		
MGDG(34:4)	768.5644	3.34	16:1-18:3
MGDG(34:2)*	772.5932	3.34	
MGDG(34:1)*	774.6076	3.34	
MGDG(36:6)	792.5622	3.34	18:3-18:3 and 18:4-18:2
MGDG(36:4)	796.5934	3.34	18:2-18:2 and 18:3-18:1
MGDG(36:3)	798.6091	3.24	18:1-18:2 and 18:3-18:0
MGDG(36:2)	800.6250	3.34	18:1-18:1
MGDG(38:1)*	830.6714	3.24	
MGDG(40:4)*	852.6562	3.24	
	**[M + NH_4_]^+^**		
DGMG(18:3)*	694.4022	3.64	18:3
DGMG(18:1)*	698.4317	3.64	18:1
	**[M + NH_4_]^+^**		
DGDG(34:4)*	930.6154	3.54	
DGDG(34:3)	932.6315	3.54	16:0-18:3
DGDG(34:2)	934.6437	3.54	16:1-18:1 and 16:0-18:2
DGDG(34:1)	936.6621	3.54	16:0-18:1
DGDG(36:6)	954.6150	3.54	18:3-18:3 and 18:4-18:2
DGDG(36:4)	958.6467	3.54	18:1-18:3 and 18:2-18:2
DGDG(36:3)	960.6582	3.54	18:2-18:1 and 18:0-18:3
DGDG(36:2)	962.6775	3.54	18:1-18:1 and 18:2-18:0
	**[M + H]^+^**		
HexCer(d34:2(OH))	714.5518	3.34	d18:2-16:0-OH
HexCer(d34:1(OH))	716.5669	3.34	d18:1-16:0-OH
HexCer(t40:1(OH))	816.6555	3.24	t18:1-22:0-OH
HexCer(t42:1(OH))	844.6874	3.24	t18:1-24:0-OH
HexCer(t44:1((OH)_2_))	872.7195	3.24	t18:1-26:0-((OH)_2_)
	**[M + H]^+^**		
MGTS(16:0)	474.3793	6.44	16:0
	**[M + H]^+^**		
DGTS(34:2)	736.6082	5.04	16:0-18:2 and 16:1-18:1
DGTS(34:1)	738.6213	5.04	16:0-18:1
DGTS(36:4)	760.6095	4.94	18:2-18:2
DGTS(36:3)	762.6238	4.94	18:2-18:1
DGTS(36:2)	764.6404	4.94	18:1-18:1

All the molecular species were identified by MS/MS, exact mass and retention time, except the molecular species with an asterisk (*) that were identified only by exact mass and by retention time. ^a^) The fatty acyl chains of these species were not identified by MS/MS since no informative negative mode spectrum could be acquired. *t*_R_, retention time. A detailed list of the polar lipid molecular species with the chemical formulas, observed and theoretical *m/z* values and mass errors are listed in Table S2 of Supplementary material.

**Table 3:** Relative abundance (in %) of the polar lipid classes identified in the olive (*Olea europaea* L. cv. *Galega vulgar*) pulp.

Relative Abundance (%)				
			Mean	S.D.
Phospholipids			71.69	2.20
PC			96.66	1.63
	Non-Hydroxylated		85.42	6.32
	Hydroxylated		14.58	6.32
LPC			2.61	1.55
	Non-Hydroxylated		97.11	1.39
	Hydroxylated		2.89	1.39
PE			0.65	0.10
SM			0.06	0.03
PG			0.01	0.00
Glycolipids			25.44	1.94
MGDG			52.29	3.14
DGDG			46.97	2.87
DGMG			0.74	0.29
Glycosphingolipids			2.33	0.81
HexCer			2.33	0.81
Betaines			0.54	0.26
DGTS			91.56	2.63
MGTS			8.44	2.63
Sum			100.00	3.32

Values represent mean ± standard deviation (S.D.) of five samples (*n* = 5) normalized to the respective internal standard used in the LC-MS. Legend: PC: phosphatidylcholines; LPC: lyso-phosphatidylcholines; PE: phosphatidylethanolamines; SM: sphingomyelins; PG: phosphatidylglycerols; MGDG: monoglycosyldiacylglycerols; DGDG: diglycosyldiacylglycerols; DGMG: diglycosylmonoacylglycerols; HexCer: hexosylceramides; DGTS: diacylglyceryl-*N,N,N*-trimethylhomoserines; MGTS: monoacylglyceryl-*N*,*N*,*N*-trimethylhomoserine.

## Supplementary Materials

The following are available online. Figure S1: Base peak chromatogram of neutral lipid-rich fraction of the olive (*Olea europaea* L. cv. *Galega vulgar*) pulp in positive-ion mode obtained by C_30_-RP-HPLC-ESI-orbitrap-MS; Table S1: List of triacylglycerols (TAG) identified in the olive (*Olea europaea* L. cv. *Galega vulgar*) pulp by C_30_-RP-HPLC-ESI-orbitrap-MS and C_30_-RP-HPLC-ESI-orbitrap-MS with the fatty acyl composition considering the different possible combinations of the fatty acids; Table S2: List of polar lipids (phospholipids, glyceroglycolipids, glycosphingolipids and betaines) identified in the olive (*Olea europaea* L. cv. *Galega vulgar*) pulp by HILIC-LC-orbitrap-MS and HILIC-LC-orbitrap-MS/MS; Figure S2: LC-MS/MS spectra of two molecular species of triacylglycerol identified in the olive (*Olea europaea* L. cv. *Galega vulgar*) pulp in the positive ion mode as [M + NH_4_]^+^ ions, bearing unusual fatty acids; Figure S3: LC-MS/MS spectra of two molecular species of phosphatidylcholine (PC) identified in the olive (*Olea europaea* L. cv. *Galega vulgar*) pulp in the negative ion mode as [M + CH_3_COO]^−^ ions, bearing unusual fatty acids.
