# Supplementary material for: Lipidomic Profiling of the Olive (Olea europaea L.) Fruit towards Its Valorisation as a Functional Food: In-Depth Identification of Triacylglycerols and Polar Lipids in Portuguese Olives
Source: Molecules. 2019 Jul 13;24(14):2555. doi: 10.3390/molecules24142555 (PMC6680557; doi:10.3390/molecules24142555)
Supplement: Supplementary file 1 [file molecules-24-02555-s001.pdf]

Supplementary Material for

Lipidomic profiling of the olive (*Olea europaea* L.) fruit towards its valorisation as a functional food: in-depth identification of triacylglycerols and polar lipids in Portuguese olives

Eliana Alves[1,*], Tânia Melo[1,2], Madalena P. Barros[3], M. Rosário M. Domingues[1,2,*], Pedro Domingues[1]

[1]Mass Spectrometry Centre, Department of Chemistry & QOPNA & LAQV-REQUIMTE, University of Aveiro, Campus Universitario de Santiago, 3810-193 Aveiro, Portugal

[2]Department of Chemistry & CESAM & ECOMARE, University of Aveiro, Campus Universitario de Santiago, 3810-193 Aveiro, Portugal

[3]Cooperativa de Olivicultores de Nelas, C.R.L., Zona Industrial de Nelas, 3520-095 Nelas, Portugal

[*] Corresponding authors: Dr. Eliana Alves E-mail: elianaalves@ua.pt; Prof. M. Rosário M. Domingues (mrd@ua.pt)

Contents

Figure S1) Base peak chromatogram of neutral lipid-rich fraction of the olive (*Olea europaea* L. cv. *Galega vulgar*) pulp in positive-ion mode obtained by C_{30}-RP-HPLC-ESI-orbitrap-MS. 3

Table S1) List of triacylglycerols (TAG) identified in the olive (*Olea europaea* L. cv. *Galega vulgar*) pulp by C_{30}-RP-HPLC-ESI-orbitrap-MS and C_{30}-RP-HPLC-ESI-orbitrap-MS with the fatty acyl composition considering the different possible combinations of the fatty acids............... 4

Table S2) List of polar lipids (phospholipids, glyceroglycolipids, glycosphingolipids and betaines) identified in the olive (*Olea europaea* L. cv. *Galega vulgar*) pulp by HILIC-LC-orbitrap-MS and HILIC-LC-orbitrap-MS/MS 10

Figure S2) LC-MS/MS spectra of two molecular species of triacylglycerol identified in the olive (*Olea europaea* L. cv. *Galega vulgar*) pulp in the positive ion mode as $[M+NH_4]^+$ ions, bearing unusual fatty acids. In this MS/MS spectrum of TAG(53:3), several neutral losses of the acyl chains as $RCOOH+NH_3$ ions are seen, including neutral losses of the unusual odd-fatty acids 15:1, 17:1 and 19:1. In this MS/MS spectrum of TAG(48:3), neutral losses of the acyl chains of unusual odd- and polyunsaturated fatty acids are seen, as 15:1 and 16:2, respectively. 15

Figure S3) LC-MS/MS spectra of two molecular species of phosphatidylcholine (PC) identified in the olive (*Olea europaea* L. cv. *Galega vulgar*) pulp in the negative ion mode as $[M+CH_3COO]^-$ ions, bearing unusual fatty acids. In the MS/MS spectrum of PC(36:3(OH)) on the left, the carboxylate anions corresponding to the fatty acids C18:1 at *m/z* 281.24, C18:2 at *m/z* 279.23, C18:2(OH) at *m/z* 295.22 and C18:1(OH) at *m/z* 297.24 are seen. In the MS/MS spectrum of PC(26:2) on the right, the carboxylate anions corresponding to the fatty acids C8:1 at *m/z* 141.09 and C18:1 at *m/z* 281.24 are seen. 16

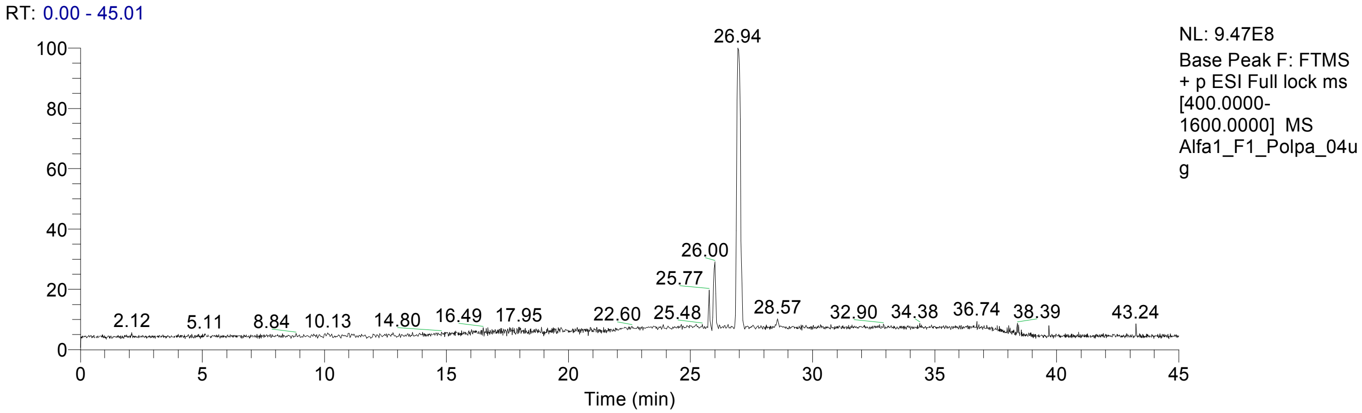

Figure S1) Base peak chromatogram of neutral lipid-rich fraction of the olive (*Olea europaea* L. cv. *Galega vulgar*) pulp in positive-ion mode obtained by C_{30}-RP-HPLC-ESI-orbitrap-MS.

Table S1) List of triacylglycerols (TAG) identified in the olive (*Olea europaea* L. cv. *Galega vulgar*) pulp by C_{30}-RP-HPLC-ESI-orbitrap-MS and C_{30}-RP-HPLC-ESI-orbitrap-MS with the fatty acyl composition considering the different possible combinations of the fatty acids

Lipid name TAG(C:N)	Fatty acyl chains	Formula $[M+NH_4]^+$	Observed Mass $[M+NH_4]^+$	Exact Mass $[M+NH_4]^+$	Mass error (ppm)
TAG(40:0)	**12:0-12:0-16:0** and 12:0-13:0-15:0 and **12:0-14:0-14:0** and 13:0-13:0-14:0	C43H86NO6	712.6446	712.6455	-1.28
TAG(41:0)	11:0-13:0-17:0 and 11:0-14:0-16:0 and 11:0-15:0-15:0 and 12:0-12:0-17:0 and 12:0-13:0-16:0 and **12:0-14:0-15:0** and 13:0-13:0-15:0 and **13:0-14:0-14:0**	C44H88NO6	726.6595	726.6612	-2.29
TAG(42:1)	**12:0-12:0-18:1** and **12:0-14:0-16:1** and 12:0-14:1-16:0 and 12:0-15:0-15:1 and 13:0-13:0-16:1 and 13:0-14:0-15:1 and 13:0-14:1-15:0 and 14:0-14:0-14:1	C45H88NO6	738.6597	738.6612	-1.98
TAG(42:0)	11:0-14:0-17:0 and 11:0-15:0-16:0 and 12:0-13:0-17:0 and **12:0-14:0-16:0** and 12:0-15:0-15:0 and 13:0-13:0-16:0 and **13:0-14:0-15:0** and 14:0-14:0-14:0	C45H90NO6	740.6758	740.6768	-1.37
TAG(43:1)	12:0-13:0-18:1 and 12:0-14:1-17:0 and 12:0-15:0-16:1 and 12:0-16:0-15:1 and and 13:0-14:1-16:0 and **13:0-14:0-16:1** and 13:0-15:0-15:1 and 14:0-14:0-15:1 and 14:0-14:1-15:0	C46H90NO6	752.6757	752.6768	-1.48
TAG(43:0)	12:0-14:0-17:0 and 12:0-15:0-16:0 and 13:0-13:0-17:0 and 13:0-14:0-16:0 and 13:0-15:0-15:0 and **14:0-14:0-15:0**	C46H92NO6	754.6914	754.6925	-1.41
TAG(44:2)	12:0-14:1-18:1 and 13:0-15:1-16:1 and 12:0-16:1-16:1 and **14:0-14:1-16:1** and 14:0-15:1-15:1 and 14:1-15:0-15:1 and 12:0-14:0-18:2	C47H90NO6	764.6763	764.6768	-0.67
TAG(44:1)	12:0-14:0-18:1 and 12:0-16:0-16:1 and 13:0-13:0-18:1 and 13:0-14:1-17:0 and 13:0-15:0-16:1 and **14:0-14:0-16:1** and 14:0-14:1-16:0 and 14:1-15:0-15:0	C47H92NO6	766.6916	766.6925	-1.13

TAG(44:0)	10:0-16:0-18:0 and 10:0-17:0-17:0 and 12:0-14:0-18:0 and 12:0-15:0-17:0 and 12:0-16:0-16:0 and 13:0-13:0-18:0 and 13:0-14:0-17:0 and 13:0-15:0-16:0 and 14:0-14:0-16:0 and **14:0-15:0-15:0**	C47H94NO6	768.7071	768.7081	-1.32
TAG(45:2)	12:0-15:1-18:1 and 13:0-14:1-18:1 and **15:0-14:1-16:1** and 15:0-15:1-15:1 and 14:1-17:0-14:1	C48H92NO6	778.6913	778.6925	-1.49
TAG(45:1)	12:0-16:0-17:1 and 13:0-15:0-17:1 and 13:0-16:0-16:1 and 14:0-14:0-17:1 and **14:0-15:0-16:1** and 14:0-15:1-16:0 and 15:0-15:0-15:1 and 14:1-15:0-16:0	C48H94NO6	780.7065	780.7081	-2.07
TAG(45:0)	12:0-15:0-18:0 and 12:0-16:0-17:0 and 13:0-14:0-18:0 and 13:0-15:0-17:0 and 13:0-16:0-16:0 and 14:0-14:0-17:0 and **14:0-15:0-16:0** and 15:0-15:0-15:0	C48H96NO6	782.7230	782.7238	-0.98
TAG(46:3)	14:1-14:1-18:1 and **14:1-16:1-16:1** and 15:1-15:1-16:1	C49H92NO6	790.6922	790.6925	-0.33
TAG(46:2)	13:0-15:1-18:1 and 13:0-16:1-17:1 and 14:0-15:1-17:1 and 14:1-15:0-17:1 and **15:0-15:1-16:1** and 15:1-15:1-16:0 and 14:0-16:1-16:1 and 14:1-16:0-16:1	C49H94NO6	792.7069	792.7081	-1.53
TAG(46:1)	14:0-14:0-18:1 and 14:0-14:1-18:0 and **14:0-16:0-16:1** and **14:1-16:0-16:0** and 15:0-15:0-16:1 and 15:0-15:1-16:0	C49H96NO6	794.7226	794.7238	-1.46
TAG(46:0)	12:0-17:0-17:0 and 12:0-16:0-18:0 and 14:0-14:0-18:0 and 14:0-15:0-17:0 and **14:0-16:0-16:0** and **15:0-15:0-16:0**	C49H98NO6	796.7386	796.7394	-1.02
TAG(47:3)*		C50H94NO6	804.7074	804.7081	-0.89
TAG(47:2)	13:0-16:1-18:1 and 13:0-17:1-17:1 and 14:0-15:0-18:2 and 14:0-15:1-18:1 and 14:0-16:1-17:1 and 14:1-15:0-18:1 and 15:0-15:1-17:1 and **15:0-16:1-16:1**	C50H96NO6	806.7227	806.7238	-1.32
TAG(47:1)	13:0-16:0-18:1 and 13:0-17:0-17:1 and 14:0-15:0-18:1 and 14:0-16:0-17:1 and 14:0-16:1-17:0 and 15:0-15:0-17:1 and 15:0-15:1-17:0 and **15:0-16:0-16:1** and 15:1-16:0-16:0	C50H98NO6	808.7380	808.7394	-1.75

TAG(47:0)	13:0-16:0-18:0 and 13:0-17:0-17:0 and 14:0-15:0-18:0 and 14:0-16:0-17:0 and 15:0-15:0-17:0 and **15:0-16:0-16:0**	C50H100NO6	810.7541	810.7551	-1.19
TAG(48:3)	12:0-18:1-18:2 and 14:0-16:2-18:1 and 14:0-16:1-18:2 and **14:1-16:1-18:1** and 14:1-17:1-17:1 and 15:0-15:1-18:2 and 15:0-16:2-17:1	C51H96NO6	818.7231	818.7238	-0.81
TAG(48:2)	14:0-16:1-18:1 and 14:1-16:0-18:1 and 15:0-15:1-18:1 and **16:0-16:1-16:1**	C51H98NO6	820.7386	820.7394	-0.99
TAG(48:1)	14:0-16:0-18:1 and 15:0-15:0-18:1 and 15:0-16:0-17:1 and **16:0-16:0-16:1**	C51H100NO6	822.7544	822.7551	-0.81
TAG(48:0)	12:0-16:0-20:0 and 12:0-17:0-19:0 and 12:0-18:0-18:0 and 13:0-15:0-20:0 and 13:0-16:0-19:0 and 13:0-17:0-18:0 and 14:0-14:0-20:0 and 14:0-15:0-19:0 and 14:0-16:0-18:0 and 14:0-17:0-17:0 and 15:0-15:0-18:0 and 15:0-16:0-17:0 and 15:0-15:0-18:0 and **16:0-16:0-16:0**	C51H102NO6	824.7700	824.7707	-0.87
TAG(49:3)	14:1-17:1-18:1 and 15:0-16:1-18:2 and 15:0-16:2-18:1 and 15:1-16:1-18:1 and 15:1-17:1-17:1 and **16:1-16:1-17:1**	C52H98NO6	832.7384	832.7394	-1.22
TAG(49:2)	14:0-17:0-18:2 and 15:0-16:0-18:2 and 15:0-16:1-18:1 and **15:1-16:0-18:1** and 15:1-17:0-17:1 and 16:1-16:1-17:0	C52H100NO6	834.7539	834.7551	-1.39
TAG(49:1)	14:0-15:0-20:1 and 14:0-16:0-19:1 and 14:0-16:1-19:0 and 14:0-17:0-18:1 and 14:0-17:1-18:0 and 15:0-15:0-19:1 and 15:0-15:1-19:0 and **15:0-16:0-18:1** and 15:0-16:1-18:0 and 15:1-16:0-18:0 and 16:0-16:0-17:1 and 16:0-16:1-17:0	C52H102NO6	836.7698	836.7707	-1.09
TAG(49:0)	14:0-16:0-19:0 and 14:0-17:0-18:0 and 15:0-15:0-19:0 and 15:0-16:0-18:0 and 15:0-17:0-17:0 and **16:0-16:0-17:0**	C52H104NO6	838.7851	838.7864	-1.51
TAG(50:4)	14:0-18:2-18:2 and 14:1-18:1-18:2 and **16:1-16:1-18:2** and 16:1-16:2-18:1	C53H98NO6	844.7388	844.7394	-0.73
TAG(50:3)	16:0-16:0-18:3 and **16:0-16:1-18:2** and 16:1-16:1-18:1	C53H100NO6	846.7541	846.7551	-1.14
TAG(50:2)	**16:0-16:1-18:1** and 14:0-18:1-18:1 and 16:1-16:1-18:0	C53H102NO6	848.7708	848.7707	0.10

TAG(50:1)	14:0-16:0-20:1 and 14:0-18:0-18:1 and **16:0-16:0-18:1** and 16:0-16:1-18:0 and 16:0-17:0-17:1 and 16:1-17:0-17:0	C53H104NO6	850.7866	850.7864	0.28
TAG(50:0)	14:0-14:0-22:0 and 14:0-16:0-20:0 and 14:0-17:0-19:0 and 14:0-18:0-18:0 and 15:0-15:0-20:0 and 15:0-16:0-19:0 and 15:0-17:0-18:0 and **16:0-16:0-18:0** and 16:0-17:0-17:0	C53H106NO6	852.8004	852.8020	-1.89
TAG(51:3)	15:0-16:1-20:2 and 15:0-17:1-19:2 and 15:0-18:1-18:2 and 15:1-16:0-20:2 and 15:1-17:0-19:2 and 15:1-18:1-18:1 and 16:0-16:1-19:2 and **16:0-17:1-18:2** and 16:0-18:1-17:2 and 17:0-17:1-17:2 and **16:1-17:0-18:2** and 17:1-17:1-17:1 and **16:1-17:1-18:1**	C54H102NO6	860.7699	860.7707	-0.95
TAG(51:2)	15:0-16:0-20:2 and 15:0-18:0-18:2 and 15:0-18:1-18:1 and 15:1-17:1-19:0 and 15:1-18:0-18:1 and **16:0-17:0-18:2** and **16:0-17:1-18:1** and 16:1-16:1-19:0 and 16:1-17:0-18:1 and 16:1-17:1-18:0 and 17:0-17:1-17:1	C54H104NO6	862.7856	862.7864	-0.89
TAG(51:1)	14:0-17:0-20:1 and 14:0-18:0-19:1 and 15:0-16:0-20:1 and 15:0-17:0-19:1 and 15:0-18:0-18:1 and 16:0-16:0-19:1 and 16:0-17:1-18:0 and and **16:0-17:0-18:1** and 16:1-17:0-18:0 and 17:0-17:0-17:1	C54H106NO6	864.8012	864.8020	-0.94
TAG(51:0)	14:0-17:0-20:0 and 14:0-18:0-19:0 and 15:0-16:0-20:0 and 15:0-17:0-19:0 and 15:0-18:0-18:0 and 16:0-16:0-19:0 and **16:0-17:0-18:0** and 17:0-17:0-17:0	C54H108NO6	866.8158	866.8177	-2.15
TAG(52:4)	16:1-18:1-18:2 and **16:0-18:2-18:2**	C55H102NO6	872.7700	872.7707	-0.82
TAG(52:3)	16:0-18:1-18:2 and **16:1-18:1-18:1** and 16:0-16:1-20:2	C55H104NO6	874.7866	874.7864	0.27
TAG(52:2)	**16:0-18:1-18:1** and 16:1-18:0-18:1	C55H106NO6	876.8027	876.8020	0.78
TAG(52:1)	16:0-16:0-20:1 and 16:1-18:0-18:0 and **16:0-18:0-18:1**	C55H108NO6	878.8169	878.8177	-0.87
TAG(52:0)	14:0-18:0-20:0 and 16:0-16:0-20:0 and **16:0-18:0-18:0**	C55H110NO6	880.8319	880.8333	-1.61
TAG(53:6)*		C56H100NO6	882.7509	882.7551	-4.72
TAG(53:4)	**17:1-18:1-18:2** and 17:2-18:1-18:1	C56H104NO6	886.7861	886.7864	-0.30

TAG(53:3)	15:1-18:1-20:1 and 15:1-19:1-19:1 and 16:0-17:1-20:2 and 16:0-18:2-19:1 and 16:1-17:0-20:2 and 16:1-17:1-20:1 and 16:1-18:1-19:1 and 17:0-18:1-18:2 and 17:1-17:1-19:1 and **17:1-18:1-18:1**	C56H106NO6	888.8013	888.8020	-0.80
TAG(53:2)	15:0-18:1-20:1 and 16:0-18:1-19:1 and 16:0-17:1-20:1 and 16:1-17:0-20:1 and 17:0-17:1-19:1 and **17:0-18:1-18:1**	C56H108NO6	890.8167	890.8177	-1.08
TAG(53:1)	15:0-17:1-21:0 and 15:0-18:0-20:1 and 15:0-18:1-20:0 and 16:0-16:1-21:0 and 16:0-17:0-20:1 and 16:0-17:1-20:0 and 16:0-18:1-19:0 and 16:1-17:0-20:0 and 16:1-18:0-19:0 and 17:0-17:1-19:0 and **17:0-18:0-18:1**	C56H110NO6	892.8316	892.8333	-1.92
TAG(54:6)	18:1-18:1-18:4 and 18:1-18:2-18:3 and **18:2-18:2-18:2**	C57H102NO6	896.7697	896.7707	-1.13
TAG(54:5)	**18:1-18:1-18:3** and 18:1-18:2-18:2	C57H104NO6	898.7859	898.7864	-0.52
TAG(54:4)	16:0-18:1-20:3 and 16:0-18:2-20:2 and 16:0-18:3-20:1 and 16:1-18:0-20:3 and 16:1-18:1-20:2 and 16:1-18:2-20:1 and 16:2-18:0-20:2 and 16:2-18:1-20:1 and 18:0-18:1-18:3 and 18:0-18:2-18:2 and **18:1-18:1-18:2**	C57H106NO6	900.8021	900.8020	0.10
TAG(54:3)	16:0-18:1-20:2 and 16:0-18:2-20:1 and 16:1-18:0-20:2 and 16:1-18:1-20:1 and 18:0-18:1-18:2 and **18:1-18:1-18:1**	C57H108NO6	902.8182	902.8177	0.59
TAG(54:2)	16:0-18:1-20:1 and **18:0-18:1-18:1** and 18:0-18:0-18:2	C57H110NO6	904.8333	904.8333	-0.02
TAG(54:1)	14:0-20:0-20:1 and 16:0-18:0-20:1 and 16:0-18:1-20:0 and 16:1-18:0-20:0 and **18:0-18:0-18:1**	C57H112NO6	906.8480	906.8490	-1.06
TAG(54:0)	14:0-15:0-25:0 and **14:0-16:0-24:0** and 14:0-18:0-22:0 and 15:0-15:0-24:0 and 15:0-18:0-21:0 and 16:0-16:0-22:0 and 18:0-18:0-18:0	C57H114NO6	908.8640	908.8646	-0.68
TAG(55:4)	18:1-18:2-19:1	C58H108NO6	914.8184	914.8177	0.80

TAG(55:2)	16:0-19:1-20:1 and 17:0-18:1-20:1 and 17:0-19:1-19:1 and 17:1-18:0-20:1 and 17:1-18:1-20:0 and 17:1-19:0-19:1 and 18:0-18:1-19:1 and **18:1-18:1-19:0**	C58H112NO6	918.8482	918.8490	-0.83
TAG(55:1)	14:0-18:1-23:0 and **16:0-18:1-21:0** and and 16:1-18:0-21:0 and 17:0-18:1-20:0	C58H114NO6	920.8642	920.8646	-0.45
TAG(56:5)	18:1-18:3-20:1	C59H108NO6	926.8155	926.8177	-2.33
TAG(56:4)	**18:1-18:1-20:2** and 18:1-18:2-20:1	C59H110NO6	928.8332	928.8333	-0.12
TAG(56:3)	18:1-18:1-20:1	C59H112NO6	930.8482	930.8490	-0.82
TAG(56:2)	16:0-20:1-20:1 and 18:0-18:1-20:1 and **18:1-18:1-20:0**	C59H114NO6	932.8640	932.8646	-0.66
TAG(56:1)	16:0-16:1-24:0 and **16:0-18:1-22:0** and 16:1-18:0-22:0 and 16:1-20:0-20:0 and **18:0-18:1-20:0**	C59H116NO6	934.8802	934.8803	-0.07
TAG(57:5)*		C60H110NO6	940.8334	940.8333	0.09
TAG(57:4)*		C60H112NO6	942.8490	942.8490	0.04
TAG(57:3)*		C60H114NO6	944.8640	944.8646	-0.65
TAG(57:2)	18:1-18:1-21:0	C60H116NO6	946.8792	946.8803	-1.12
TAG(58:3)	18:1-18:2-22:0	C61H116NO6	958.8793	958.8803	-1.01
TAG(58:2)	18:1-18:1-22:0	C61H118NO6	960.8956	960.8959	-0.33
TAG(58:1)	16:0-16:1-26:0 and **16:0-18:1-24:0** and 18:0-18:1-22:0 and 18:1-20:0-20:0	C61H120NO6	962.9106	962.9116	-1.00
TAG(60:3)	18:1-18:2-24:0	C63H120NO6	986.9110	986.9116	-0.57
TAG(60:2)	18:1-18:1-24:0	C63H122NO6	988.9265	988.9272	-0.72

C:N means total number of carbons (C) and number of double bonds (N). * means that species were confirmed by exact mass in the MS without MS/MS confirmation. The fatty acyl compositions in **bold** letters mean the major TAG species for the same *m/z* based on the abundance of the fragment ions in the MS/MS.

Table S2) List of polar lipids (phospholipids, glyceroglycolipids, glycosphingolipids and betaines) identified in the olive (*Olea europaea* L. cv. *Galega vulgar*) pulp by HILIC-LC-orbitrap-MS and HILIC-LC-orbitrap-MS/MS

Lipid name Class(C:N)	Acyl chain composition	Formula $[M+H]^+$	Observed Mass $[M+H]^+$	Exact Mass $[M+H]^+$	Mass error (ppm)
PC(24:0(OH)	16:0-8:0(OH)	C32H65NO9P	638.4399	638.4397	0.31
PC(26:2)	8:1-18:1	C34H65NO8P	646.4436	646.4448	-1.86
PC (16:0/C8CHO)*	16:0/C8CHO	C33H65NO9P	650.4400	650.4397	0.47
PC(26:2(OH))	18:1-8:1(OH)	C34H65NO9P	662.4403	662.4397	0.91
PC(26:1(OH))	18:1-8:0(OH)	C34H67NO9P	664.4585	664.4553	4.82
PC (16:0/C8COOH)*	16:0/C8COOH	C33H65NO10P	666.4348	666.4346	0.28
PC(27:2(OH))	18:1-9:1(OH)	C35H67NO9P	676.4560	676.4553	1.03
PC(28:3(OH))	18:1-10:2(OH)	C36H67NO9P	688.4554	688.4553	0.15
PC(30:3)[a)]	30:03:00	C38H71NO8P	700.4895	700.4917	-3.14
PC(29:2(OH))	18:1-11:1(OH)	C37H71NO9P	704.4867	704.4866	0.14
PC(32:2)	16:1-16:1	C40H77NO8P	730.5379	730.5387	-1.10
PC(32:1)	16:0-16:1 and 14:0-18:1	C40H79NO8P	732.5539	732.5543	-0.55
PC(32:2(OH))	16:0-16:2(OH) and 16:1-16:1(OH) and 18:1-14:1(OH)	C40H77NO9P	746.5316	746.5336	-2.68
PC(33:1)	16:0-17:1	C41H81NO8P	746.5671	746.5700	-3.88
PC(32:1(OH))	18:1-14:0(OH) and 16:0-16:1(OH)	C40H79NO9P	748.5476	748.5492	-2.20
PC(34:4)	16:1-18:3	C42H77NO8P	754.5372	754.5387	-1.99
PC(34:3)	16:0-18:3 and 16:1-18:2	C42H79NO8P	756.5545	756.5543	0.26
PC(34:2)	16:0-18:2 and 16:1-18:1	C42H81NO8P	758.5699	758.5700	-0.13
PC(34:1)	16:0-18:1	C42H83NO8P	760.5854	760.5856	-0.26
PC(34:4(OH))	17:1-17:3(OH) and 18:1-16:3(OH) and 18:2-16:2(OH)	C42H77NO9P	770.5338	770.5336	0.26
PC(35:3)	18:2-17:1	C43H81NO8P	770.5697	770.5700	-0.39

PC(35:2)	18:1-17:1	C43H83NO8P	772.5848	772.5856	-1.04
PC(34:3(OH))	16:0-18:3(OH) and 16:1-18:2(OH) and 18:1-16:2(OH)	C42H79NO9P	772.5487	772.5492	-0.65
PC(34:2(OH))	16:1-18:1(OH) and 16:0-18:2(OH) and 18:1-16:1(OH)	C42H81NO9P	774.5646	774.5649	-0.39
PC(35:1)	18:1-17:0	C43H85NO8P	774.5998	774.6013	-1.94
PC(34:1(OH))	16:0-18:1(OH)	C42H83NO9P	776.5799	776.5805	-0.77
PC(36:6)	18:3-18:3	C44H77NO8P	778.538	778.5387	-0.90
PC(36:5)	18:3-18:2	C44H79NO8P	780.5512	780.5543	-3.97
PC(36:4)	18:2-18:2 and 18:3-18:1	C44H81NO8P	782.5689	782.567	2.43
PC(36:3)	18:1-18:2	C44H83NO8P	784.5853	784.5856	-0.38
PC(36:2)	18:1-18:1	C44H85NO8P	786.6009	786.6013	-0.51
PC(34:1(2OH))	16:0-18:1(2OH)	C42H83NO10P	792.5756	792.5755	0.13
PC(36:5(OH))	18:2-18:3(OH)	C44H79NO9P	796.5476	796.5492	-2.01
PC(37:4)	18:1-19:3 and 18:2-19:2	C45H83NO8P	796.5829	796.5856	-3.39
PC(36:4(OH))	18:1-18:3(OH) and 18:2-18:2(OH)	C44H81NO9P	798.5647	798.5649	-0.25
PC(36:3(OH))	18:1-18:2(OH) and 18:2-18:1(OH)	C44H83NO9P	800.5798	800.5805	-0.87
PC(37:2)	19:0-18:2 and 18:1-19:1	C45H87NO8P	800.6156	800.6169	-1.62
PC(36:2(OH))	18:1-18:1(OH)	C44H85NO9P	802.5949	802.5962	-1.62
PC(38:3)	18:1-20:2 and 18:2-20:1	C46H87NO8P	812.614	812.6169	-3.57
PC(38:2)	18:1-20:1	C46H89NO8P	814.6318	814.6326	-0.98
PC(38:1)	18:1-20:0	C46H91NO8P	816.6452	816.6482	-3.67
PC(36:3(2OH))	18:1-18:2(2OH)	C44H83NO10P	816.5755	816.5755	0.00
PC(36:2(2OH))	18:1-18:1(2OH)	C44H85NO10P	818.5908	818.5911	-0.37
PC(40:2)	18:1-22:1 and 18:2-22:0	C48H93NO8P	842.6611	842.6639	-3.32
PC(40:1)	18:1-22:0	C48H95NO8P	844.6812	844.6795	2.01
PC(41:1)	18:1-23:0	C49H97NO8P	858.6943	858.6952	-1.05
PC(42:1)	18:1-24:0	C50H99NO8P	872.7111	872.7108	0.34

PC(43:1)	18:1-25:0	C51H101NO8P	886.7261	886.7265	-0.45
		[M+H]+	[M+H]+	[M+H]+	
LPC(16:1)	16:1	C24H49NO7P	494.3245	494.3247	-0.40
LPC(16:0)	16:0	C24H51NO7P	496.3402	496.3403	-0.20
LPC(18:3)	18:3	C26H49NO7P	518.3224	518.3247	-4.44
LPC(18:2)	18:2	C26H51NO7P	520.3404	520.3403	0.19
LPC(18:1)	18:1	C26H53NO7P	522.3559	522.3560	-0.19
LPC(18:2(OH))	18:2(OH)	C26H51NO8P	536.3355	536.3352	0.56
LPC(18:1(OH))	18:1(OH)	C26H53NO8P	538.3511	538.3509	0.37
LPC(20:1)	20:1	C28H57NO7P	550.3876	550.3873	0.61
LPC(20:0)	20:0	C28H59NO7P	552.4025	552.4029	-0.75
LPC(18:1(2OH))	18:1(2OH)	C26H53NO9P	554.3457	554.3458	-0.18
LPC(22:0)	22:0	C30H63NO7P	580.4359	580.4342	2.90
LPC(24:0)	24:0	C32H67NO7P	608.4664	608.4655	1.45
		[M+H]+	[M+H]+	[M+H]+	
PE(30:3)[a)]		C35H65NO8P	658.4426	658.4448	-3.31
PE(34:2)	16:1-18:1 and 16:0-18:2	C39H75NO8P	716.5216	716.5230	-2.00
PE(34:1)	16:0-18:1	C39H77NO8P	718.5380	718.5387	-0.97
PE(36:4)*		C41H75NO8P	740.5216	740.5230	-1.93
PE(36:3)	18:1-18:2	C41H77NO8P	742.5388	742.5387	0.13
PE(36:2)	18:1-18:1 and 18:0-18:2 and 17:1-19:1	C41H79NO8P	744.5538	744.5543	-0.67
		[M-H]-	[M-H]-	[M-H]-	
PG(34:1)	16:0-18:1	C40H76O10P	747.5175	747.5176	-0.13
PG(36:2)*		C42H78O10P	773.5332	773.5333	-0.13
		[M+H]+	[M+H]+	[M+H]+	
SM(d34:1)		C39H80N2O6P	703.5755	703.5754	0.14

SM(d36:1)		C41H84N2O6P	731.6054	731.6067	-1.78
SM(t38:1)*		C43H88N2O7P	775.6330	775.6329	0.13
SM(d41:1)		C46H94N2O6P	801.6875	801.6850	3.12
SM(t40:0)		C45H94N2O7P	805.6792	805.6799	-0.87
SM(d42:2)		C47H94N2O6P	813.6870	813.6850	2.52
SM(d42:1)		C47H96N2O6P	815.6969	815.7006	-4.54
SM(t41:1)*		C46H94N2O7P	817.6834	817.6799	4.28
SM(t41:0)		C46H96N2O7P	819.6953	819.6955	-0.24
		$[M+NH_4]^+$	$[M+NH_4]^+$	$[M+NH_4]^+$	
MGDG(34:4)	16:1-18:3	C43H78NO10	768.5644	768.5626	2.34
MGDG(34:2)*		C43H82NO10	772.5932	772.5939	-0.87
MGDG(34:1)*		C43H84NO10	774.6076	774.6095	-2.48
MGDG(36:6)	18:3-18:3 and 18:4-18:2	C45H78NO10	792.5622	792.5626	-0.50
MGDG(36:4)	18:2-18:2 and 18:3-18:1	C45H82NO10	796.5934	796.5939	-0.63
MGDG(36:3)	18:1-18:2 and 18:3-18:0	C45H84NO10	798.6091	798.6095	-0.50
MGDG(36:2)	18:1-18:1	C45H86NO10	800.6250	800.6251	-0.15
MGDG(38:1)*		C47H92NO10	830.6714	830.6721	-0.87
MGDG(40:4)*		C49H90NO10	852.6562	852.6565	-0.32
		$[M+NH_4]^+$	$[M+NH_4]^+$	$[M+NH_4]^+$	
DGMG(18:3)*	18:03	C33H60NO14	694.4022	694.4014	1.18
DGMG(18:1)*	18:01	C33H64NO14	698.4317	698.4327	-1.41
		$[M+NH_4]$+	$[M+NH_4]$+	$[M+NH_4]$+	
DGDG(34:4)*		C49H88NO15	930.6154	930.6154	0.00
DGDG(34:3)	16:0-18:3	C49H90NO15	932.6315	932.6310	0.54
DGDG(34:2)	16:1-18:1 and 16:0-18:2	C49H92NO15	934.6437	934.6467	-3.21
DGDG(34:1)	16:0-18:1	C49H94NO15	936.6621	936.6623	-0.21

DGDG(36:6)	18:3-18:3 and 18:4-18:2	C51H88NO15	954.6150	954.6154	-0.42
DGDG(36:4)	18:1-18:3 and 18:2-18:2	C51H92NO15	958.6467	958.6467	0.00
DGDG(36:3)	18:2-18:1 and 18:0-18:3	C51H94NO15	960.6582	960.6623	-4.27
DGDG(36:2)	18:1-18:1 and 18:2-18:0	C51H96NO15	962.6775	962.6780	-0.52
		$[M+H]^+$	$[M+H]^+$	$[M+H]^+$	
HexCer(d34:2(OH))	d18:2-16:0-OH	C40H76NO9	714.5518	714.5520	-0.28
HexCer(d34:1(OH))	d18:1-16:0-OH	C40H78NO9	716.5669	716.5676	-0.98
HexCer(t40:1(OH))	t18:1-22:0-OH	C46H90NO10	816.6555	816.6565	-1.22
HexCer(t42:1(OH))	t18:1-24:0-OH	C48H94NO10	844.6874	844.6878	-0.47
HexCer(t44:1($(OH)_2$))	t18:1-26:0-($(OH)_2$)	C50H98NO10	872.7195	872.7191	0.49
		$[M+H]^+$	$[M+H]^+$	$[M+H]^+$	
MGTS(16:0)	16:0	C26H52O6N	474.3793	474.3795	-0.42
		$[M+H]^+$	$[M+H]^+$	$[M+H]^+$	
DGTS(34:2)	16:0-18:2 and 16:1-18:1	C44H82O7N	736.6082	736.6091	-1.22
DGTS(34:1)	16:0-18:1	C44H84O7N	738.6213	738.6248	-4.74
DGTS(36:4)	18:2-18:2	C46H82O7N	760.6095	760.6091	0.53
DGTS(36:3)	18:2-18:1	C46H84O7N	762.6238	762.6248	-1.31
DGTS(36:2)	18:1-18:1	C46H86O7N	764.6404	764.6404	0.00

Abbreviations: PC, phosphatidylcholine; LPC, lyso-phosphatidylcholine; PE, phosphatidylethanolamine; PG, phosphatidylglycerol; SM: sphingomyelin; MGDG, monoglycosyldiacylglycerol; DGMG, diglycosylmonoacylglycerol; DGDG, diglycosyldiacylglycerol; HexCer, hexosylceramide; MGTS, monoacylglyceryl-N,N,N-trimethylhomoserine; DGTS, diacylglyceryl-N,N,N-trimethylhomoserine.

TAG(53:3)

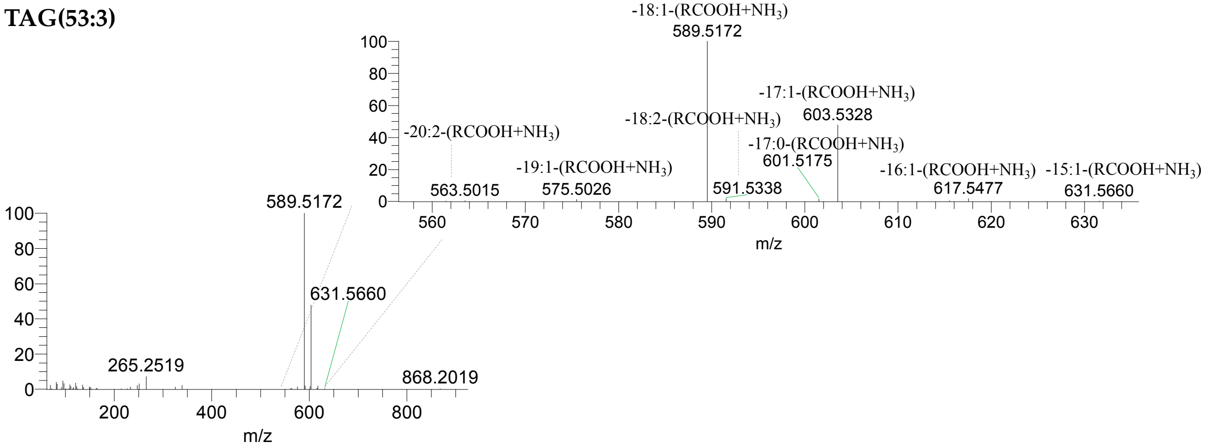

TAG(48:3)

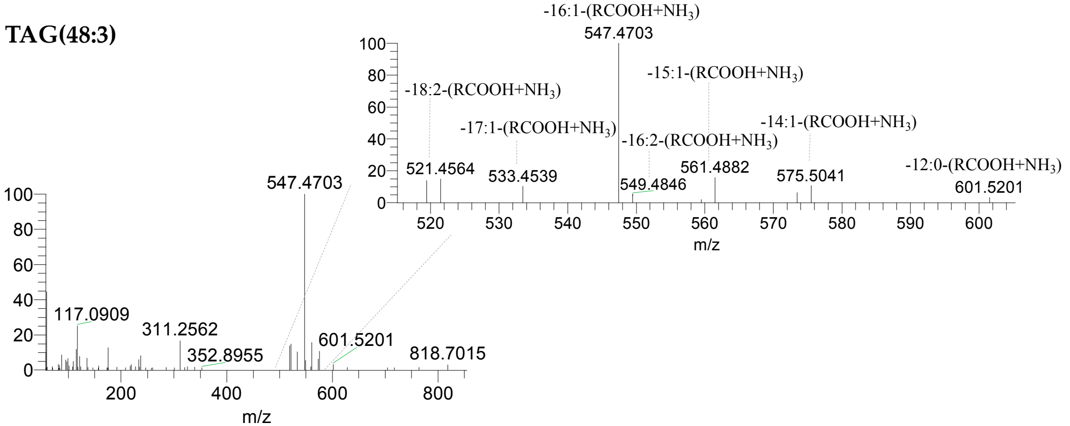

Figure S2) LC-MS/MS spectra of two molecular species of triacylglycerol identified in the olive (*Olea europaea* L. cv. *Galega vulgar*) pulp in the positive ion mode as $[M+NH_4]^+$ ions, bearing unusual fatty acids. In this MS/MS spectrum of TAG(53:3), several neutral losses of the acyl chains as $RCOOH+NH_3$ ions are seen, including neutral losses of the unusual odd-fatty acids 15:1, 17:1 and 19:1. In this MS/MS spectrum of TAG(48:3), neutral losses of the acyl chains of unusual odd- and polyunsaturated fatty acids are seen, as 15:1 and 16:2, respectively.

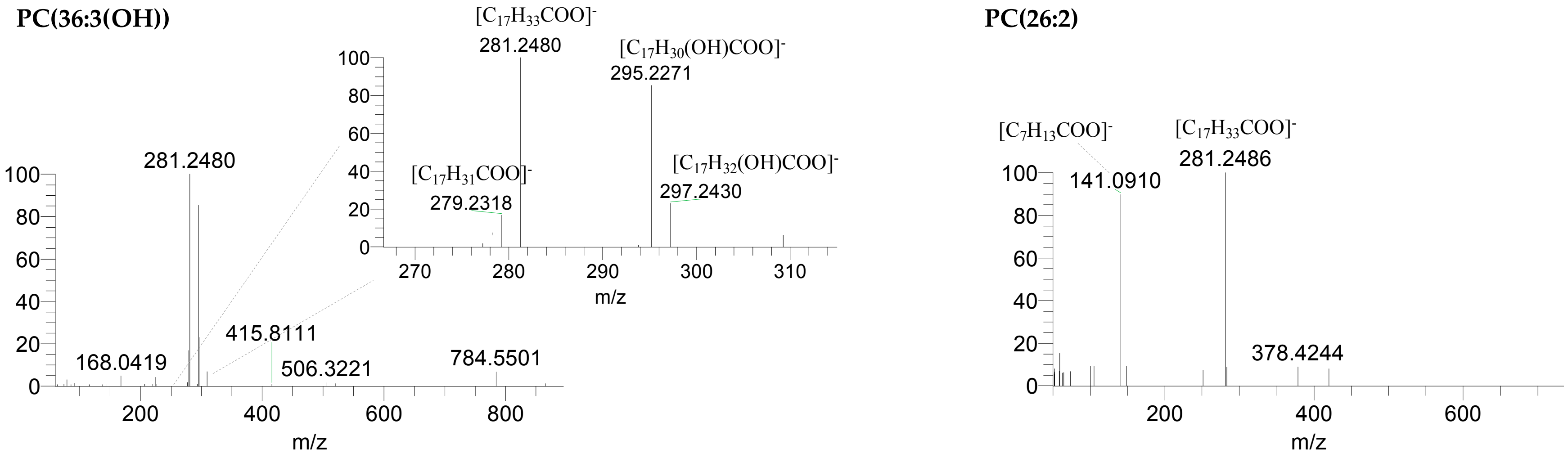

Figure S3) LC-MS/MS spectra of two molecular species of phosphatidylcholine (PC) identified in the olive (*Olea europapea* L. cv. *Galega vulgar*) pulp in the negative ion mode as $[M+CH_3COO]^-$ ions, bearing unusual fatty acids. In the MS/MS spectrum of PC(36:3(OH)) on the left, the carboxylate anions corresponding to the fatty acids C18:1 at *m/z* 281.24, C18:2 at *m/z* 279.23, C18:2(OH) at *m/z* 295.22 and C18:1(OH) at *m/z* 297.24 are seen. In the MS/MS spectrum of PC(26:2) on the right, the carboxylate anions corresponding to the fatty acids C8:1 at *m/z* 141.09 and C18:1 at *m/z* 281.24 are seen.
